# Establishment of a Prognostic Model of Lung Adenocarcinoma Based on Tumor Heterogeneity

**DOI:** 10.3389/fmolb.2022.807497

**Published:** 2022-04-11

**Authors:** Pengdou Zheng, Huojun Zhang, Weiling Jiang, Lingling Wang, Lu Liu, Yuhao Zhou, Ling Zhou, Huiguo Liu

**Affiliations:** Department of Respiratory and Critical Care Medicine, Key Laboratory of Pulmonary Diseases of Health Ministry, Tongji Hospital, Tongji Medical College, Huazhong University of Science and Technology, Wuhan, China

**Keywords:** LUAD, prognosis model, tumor microenvironment, tumor differentiation, CCL20

## Abstract

Lung cancer is one of the main cancer types due to its persistently high incidence and mortality, yet a simple and effective prognostic model is still lacking. This study aimed to identify independent prognostic genes related to the heterogeneity of lung adenocarcinoma (LUAD), generate a prognostic risk score model, and construct a nomogram in combination with other pathological characteristics to predict patients’ overall survival (OS). A significant amount of data pertaining to single-cell RNA sequencing (scRNA-seq), RNA sequencing (RNA-seq), and somatic mutation were used for data mining. After statistical analyses, a risk scoring model was established based on eight independent prognostic genes, and the OS of high-risk patients was significantly lower than that of low-risk patients. Interestingly, high-risk patients were more sensitive and effective to immune checkpoint blocking therapy. In addition, it was noteworthy that CCL20 not only affected prognosis and differentiation of LUAD but also led to poor histologic grade of tumor cells. Ultimately, combining risk score, clinicopathological information, and CCL20 mutation status, a nomogram with good predictive performance and high accuracy was established. In short, our research established a prognostic model that could be used to guide clinical practice based on the constantly updated big multi-omics data. Finally, this analysis revealed that CCL20 may become a potential therapeutic target for LUAD.

## Introduction

Lung cancer has been one of the most leading causes of cancer death worldwide. According to data from the American Cancer Society in 2021, there are approximately 235,760 new cases and 131,880 deaths, and lung cancer ranks second and first in morbidity and mortality, respectively, among malignant tumors (Siegel et al., 2021). Based on histological subtypes, lung cancer is divided into small cell lung cancer and non-small cell lung cancer (NSCLC). NSCLC represents 85% of all lung cancers and includes lung adenocarcinoma (LUAD), lung squamous cell carcinoma, and large cell carcinoma. Among them, LUAD is the largest NSCLC subgroup, accounting for 40% of all lung tumors (Denisenko et al., 2018). Despite advances in surgical resection, radiotherapy, chemotherapy, immunotherapy, and targeted therapy over the past decades, the prognosis of patients with LUAD is still very poor, with a 5-year survival rate of 15% (Herbst et al., 2018; Zhang et al., 2020a; Isla et al., 2020; Ruiz-Cordero and Devine, 2020). Therefore, it is necessary to explore this condition at the molecular level to enable accurate diagnosis, treatment, and prognosis assessments.

It is a scientific consensus that tumors are highly heterogeneous (de Sousa and Carvalho, 2018). Heterogeneity makes sensitive cells die as a result of the drug’s cytotoxicity, whereas drug-resistant cells survive due to mutations, gene expression changes, alternative splicing, etc., resulting in a poor prognosis of patients and also leading to different sensitivity levels and subsequently different treatment schemes (Aleksakhina et al., 18722019; Dagogo-Jack and Shaw, 2018; Vasan et al., 2019; Marusyk et al., 2020; Narayanan et al., 2020). Therefore, an accurate understanding of tumor heterogeneity is of great significance for assessing the prognosis and for selecting a reasonable and feasible treatment plan. To some extent, the emergence of heterogeneity can be attributed to the evolution of tumors (Lim and Ma, 2019). Single-cell transcriptomics analysis provides novel ideas for solving this problem. The Monocle 2 algorithm uses the reverse graph embedding method to project high-dimensional data into a low-dimensional space, learn cell trajectories, and different cell evolution according to pseudo-time values, thereby successfully constructing differentiation trajectories (Trapnell et al., 2014; Qiu et al., 2017a; Qiu et al., 2017b). Monocle2 has been successfully applied to many types of cancers, such as hepatocellular carcinoma, glioblastoma, multiple myeloma, laryngeal carcinoma, etc., thus verifying the authority, practicability, and reliability of the algorithm (Liang et al., 2020; Ionkina et al., 2021). Unfortunately, monocle2 has not been widely used to screen prognostic factors associated with LUAD.

Considering this issue from a micro perspective, the heterogeneity of tumors can also be partially explained by the tumor microenvironment. Malignant solid tumor tissues include tumor cells but also other types of cells, such as stromal cells, immune cells, vascular cells, etc. (Wu and Dai, 2017). A series of studies have reported that immune cells have a complex impact on tumor growth, disease progression, and drug resistance (Wang et al., 2019a; Greten and Grivennikov, 2019; Hinshaw and Shevde, 2019). Interestingly, recent studies have shown that tumor purity, which represents the proportion of tumor cells in all cells residing in the tumor microenvironment, is related to prognosis. These studies consider that low tumor purity will cause tumor immune escape, which is associated to unfavorable prognosis (Zhang et al., 2017; Gong et al., 2020). In this article, the ESTIMATE (Yoshihara et al., 2013) and CIBERSORT (Newman et al., 2015) algorithms can help us better understand the influence of various immune components and tumor purity on the prognosis of LUAD.

Aiming at the heterogeneity of tumors, many studies had attempted to establish LUAD prognosis prediction models from different aspects such as immune cells, ferroptosis-related genes, non-coding RNAs, and glycolysis genes, among which immune-related prognosis studies accounted for the majority (Liu et al., 2019; Li et al., 2020; Zuo et al., 2020; Gao et al., 2021; Zheng et al., 2021). For example, Yuwei Zhang et al. noted the correlation between tumor invasion environment, especially tumor immune system, and tumor progression and treatment outcome, and constructed a 27-gene prognostic model based on multiple omics data (Zhang et al., 2020b). Chenghan Luo et al. trained a prognostic model based on two immune genes, ANLN and F2, by using information from 761 LUAD patients (Luo et al., 2020). Both studies noted the effect of tumor immune factors on patient prognosis, and constructed relatively satisfactory models with concordance indexes of 0.73 and 0.66, respectively. However, the analysis of these studies was still incomplete, factors such as tumor purity and the proportion of immune cells had not been taken into account, and the models still needed to be further improved. This study aims to explore the independent prognostic risk genes associated with heterogeneity in LUAD and to construct a prognostic model in combination with other basic clinical information that is capable of predicting the corresponding possible immunotherapy responses of high- and low-risk patients.

## Materials and Methods

### Acquisition, Dimensionality Reduction and Cell Annotation of scRNA-Seq Data

The scRNA-seq data of three human primary LUAD samples with a reading depth of 10× genomics based on Illumina NextSeq 500, accession numbers GSM3304007, GSM3304011 and GSM3304013, were downloaded from the Gene Expression Omnibus (GEO, http://www.ncbi.nlm.nih.gov/geo/) database. After that, the scRNA-seq data was preliminarily filtered through the Seurat package in order to obtain higher quality cells. The cells and genes used for subsequent analysis needed to meet the following three quality control standards: 1) the gene was detected in at least three cells, 2) the cell had at least 50 genes detected, and 3) mitochondrial genes accounted for no more than 5%. The FeatureScatter function was run to analyze the correlation between the sequencing depth and total intracellular sequences. The data was normalized by LogNormalize and 1,500 highly variable genes among cells were selected by the default parameter vst for subsequent cell type identification. Principal component analysis (PCA), a multivariate statistical analysis method, was picked for preliminary linear dimensionality reduction (Lall et al., 2018). Each principal components (PC) essentially represented a “meta feature”, which combined relatively concentrated information of related features. Therefore, the PC at the top is more likely to represent the data set. In this study, we chose the first 15 PCs with a *p* value < 0.05 and further analyzed them through the non-linear dimensionality reduction technique the t-distributed stochastic neighbor embedding (t-SNE) algorithm to obtain the main clusters (Satija et al., 2015). With adjusted *p* value < 0.05 and | log2[fold change (FC)] | > 1 as the cutoff criterion, FindAllMarkers command was used to find the representative genes of each cluster. Finally, SingleR package was run to automatically annotate the corresponding cell type of each cluster according to the marker gene.

### Pseudo-Time Analysis and Enrichment Analysis of Lung Adenocarcinoma Differentiation-Related Genes Base on Single-Cell RNA Sequencing Data

We used an unsupervised method to select genes and ran the Monocle2 algorithm to perform non-linear dimensionality reduction, that was, to project the obtained high-dimensional data matrix into a low-dimensional space (Qiu et al., 2017a). The de-embedding machine learning algorithm learned and constructed cell trajectories, and finally projected all cells onto a root and several main branches. Different branches corresponded to different directions of differentiation, and cells on the same branch were considered to have the same differentiation characteristics. Using adjusted *p* value < 0.05 and | log2FC] | > 1 as the significance standard, the significantly differentially expressed genes among the main branches and the root were defined as LUAD differentiation-related genes (LDRGs). WEB-based GEne SeT AnaLysis Toolkit (WebGestalt, http://www.webgestalt.org/) was used for geneontology [GO, including cellular component (CC), biological process (BP), molecular function (MF)] and kyoto encyclopedia of genes and genomes (KEGG) analysis.

### Acquisition and Processing of RNA Sequencing Data

RNA-seq data of LUAD samples from GEO and TCGA databases were used in this study. The RNA-seq data matrix composed of 443 LUAD samples was obtained from GSE68465 in the GEO (http://www.ncbi.nlm.nih.gov/geo/) database. This cohort was used for 1) molecular classification, 2) analysis of the correlation between prognosis and clinicopathological characteristics, and between prognosis and tumor microenvironment, and 3) verification of prognostic models. The RNA-seq data matrix composed of 535 LUAD samples and 59 normal samples was downloaded from the Cancer Genome Atlas (TCGA, https://portal.gdc.cancer.gov/) database for the construction of prognostic models.

### Molecular Subtype Classification of Lung Adenocarcinoma Patients Based on Lung Adenocarcinoma Differentiation-Related Genes

After preprocessing, log2-scale transformation and normalization of the RNA-seq matrix, the expression data of LDRGs were extracted for the following consistent clustering analysis. ConsensusClusterPlus, an R package based on resampling methods to verify the rationality of clustering and evaluate the stability of clusters, was used to perform consensus clustering on LUAD samples. The main parameters were 50 re-sampling, 0.8 sample sampling ratio, max K = 9, “pam” clustering algorithm and euclidean distance algorithm. The cumulative distribution function (CDF) curve helps researchers determine the number of subtypes in a visual form. Kaplan-Meier analysis was chosen to compare survival differences between different clusters.

### Tumor Microenvironment Scores, Immune Cell Infiltration and Immune Checkpoint Gene Expression Across Molecular Subtypes

The study calculated the immune/stromal scores and tumor purity of each sample through the ESTIMATE package (Yoshihara et al., 2013). The content of 22 immune cells in each sample from GSE68465 was identified by CIBERSORT package (Newman et al., 2015). Meanwhile, the infiltration density of immune cells and expression of 30 validated ICGs in different LUAD subtypes was compared by the limma package. Kaplan-Meier analysis was used to investigate the prognostic value of immune cells and ICGs。

### Risk Scoring Model Generation and Validation

TCGA cohort was used as the training set to generate the risk scoring model, and GSE68465 dataset was used as the test set to verify the model. The expression data of LDRGs, poorly histological grade-related genes and ICGs in the training set and testing set were extracted, and the data were normalized and corrected by log2-scale transformation. Genes with independent prognostic performance were screened by univariate analysis, LASSO regression and multivariate Cox analysis to generate a risk scoring model. The risk scoring could be calculated as the sum of the products of gene expression levels and coefficients. The patients were divided into high-risk groups and low-risk groups based on the mean value of the risk scores. Kaplan-Meier analysis and ROC curves were completed to evaluate model accuracy and predictive performance.

### Establishment and Validation of a Nomogram Based on the The Cancer Genome Atlas Cohort

Based on TCGA cohort, age, gender, tumor purity, stage, risk score and CCL20 mutation status were included in univariate and multivariate analyses, respectively. Subsequently, independent prognostic variables were combined into nomograms to predict OS at 1-, 3-, and 5-year. ROC curves and calibration curves were used to evaluate the predictive performance and accuracy of the nomogram.

### Immunotherapy Response

The tumor immune dysfunction and elimination (TIDE, http://tide.dfci.harvard.edu/) is a computational method that evaluates the prognostic effect of immune checkpoint blocking therapy. It is used in our study to determine the response of high- and low-risk LUAD patients to immunotherapy.

### Statistical Analysis and Data Visualization

Statistical analysis and data visualization used R software (version 4.0.4) and Perl (version 5.28.1.0). Packages such as ggplot2, limma, edgeR, survival and venn. diagram in R software are used for data analysis and visualization.

## Results

### Ten Cell Clusters Were Identified and Annotated Based on Marker Genes

**FIGURE 1:**
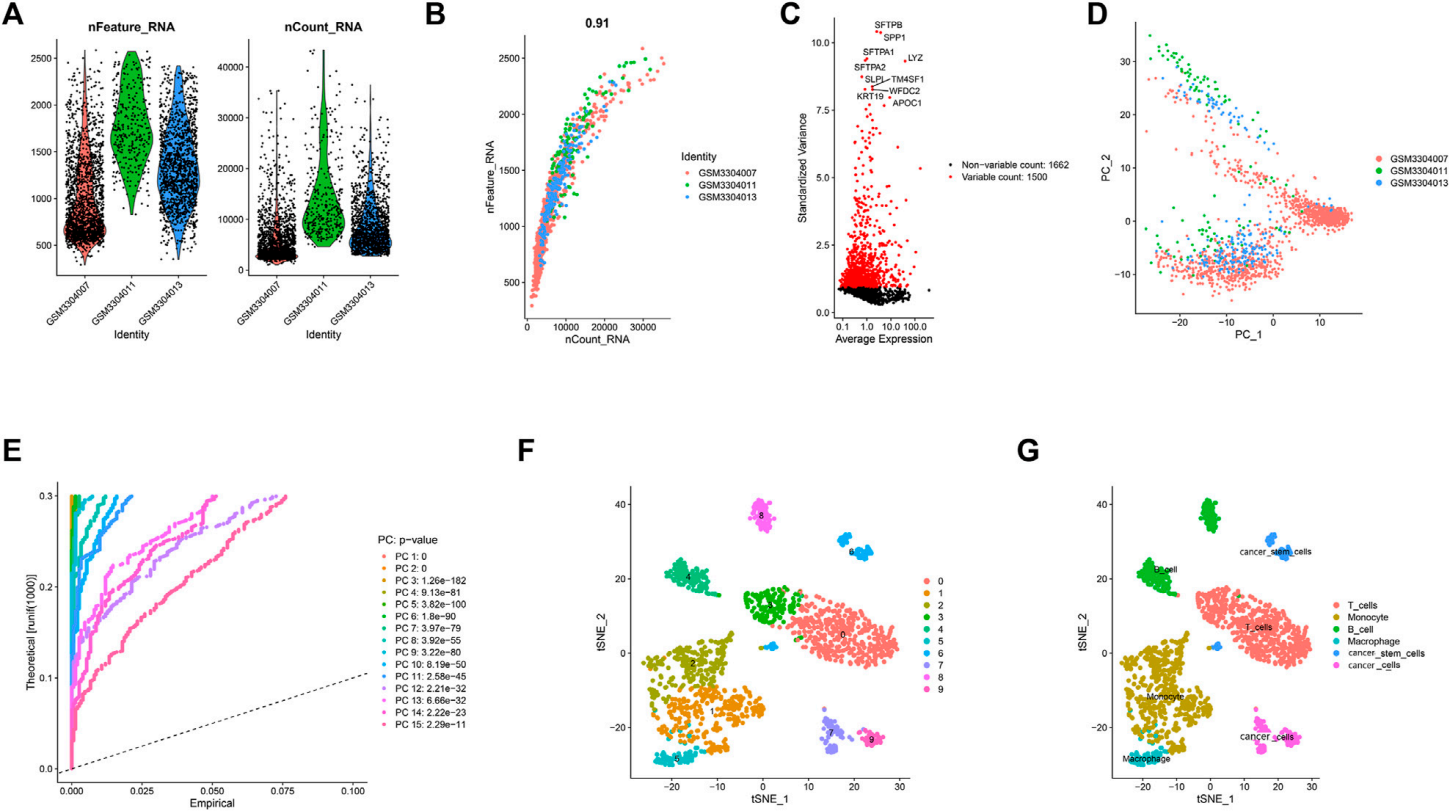
10 cell clusters were identified and annotated based on marker genes. **(A)** After quality control of the scRNA-seq data, 3,583 cells from the tumor cores of three human LUAD samples were included in the analysis. **(B)** Correlation analysis between sequencing depth and detected genes was examined, and the Pearson correlation coefficient was 0.91. **(C)** The variance of 3,162 genes were analyzed, the results indicated that 1,500 genes marked with the red dots had high variation and 1,662 genes marked with the black dots had low variation. **(D)** PCA showed no significant separations of cells in LUAD. **(E)** PCA determined 15 PCs with an estimated *p* value < 0.01. **(F)** According to t-SNE algorithm, 1695 LUAD cells were successfully aggregated into 10 clusters. **(G)** Based on the marker genes, 10 clusters were annotated by the singleR.

After quality control and normalization of LUAD scRNA-seq data, 1,695 high-quality cells and 3,162 genes from LUAD cores were included for subsequent analysis in this study (Figure 1A). The number of genes detected was obviously positively correlated with the depth of sequencing (Figure 1B). To better distinguish cell types, we used variance analysis to identify the 1,500 highly variable genes (Figure 1C). Furthermore, PCA was used to reduce data dimensionality and screen related genes. The top 20 related genes from PCs are shown as dot plots and heatmaps in the Supplementary Figure S1. However, the PCA results found that there were no clear separations among LUAD cells (Figure 1D). Therefore, we selected 15 primary PCs with significant differences (*p* values < 0.01) for further analysis (Figure 1E). Based on t-SNE algorithm, 1,695 LUAD cells were successfully divided into ten clusters (Figure 1F), and the application of differential analysis revealed 1,693 marker genes (Supplementary Figure S2). According to the marker genes, these clusters were annotated by singleR (Figure 1G). A total of 566 cells in clusters 0 and three were annotated as T cells, 598 cells in clusters 1 and 2 as monocytes, 216 cells in clusters 4 and 8 as B cells, 90 cells in cluster 5 as macrophages, 87 cells in cluster 6 as cancer stem cells, and 138 cells in clusters 7 and 9 as cancer cells.

### Trajectory Analysis Identified Two Branches, and Gene Set Enrichment Analysis Analyzed Biological Functions

**FIGURE 2:**
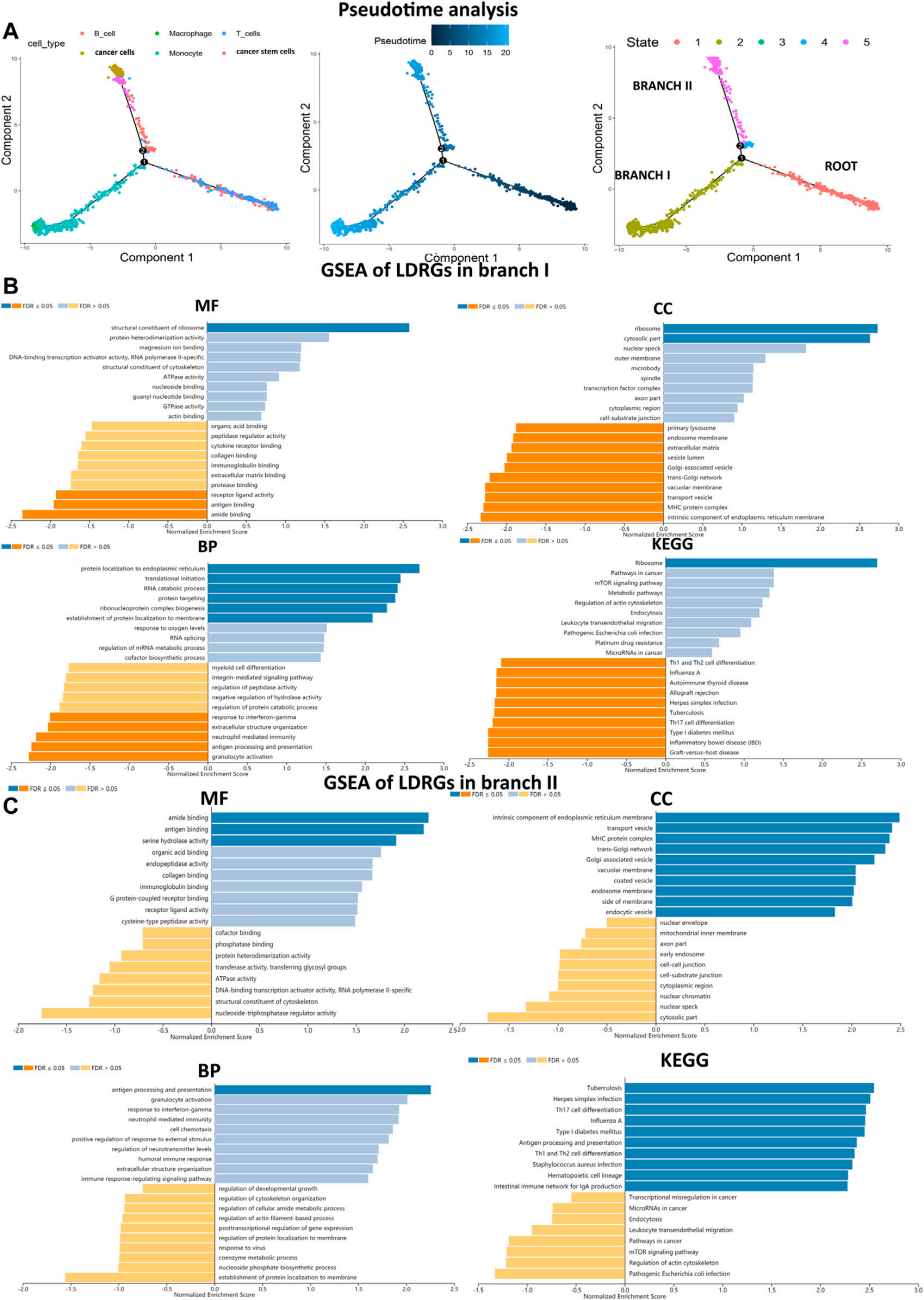
Trajectory analysis identified two branches, and GSEA analyzed biological functions. **(A)** Pseudo-time analysis reveals two LUAD branches with different evolution trajectories. **(B,C)** MF, CC, BP and KEGG for branches I and II LDRGs were conducted to assess related biological functions. An FDR < 0.05 was considered statistically significant. Blue represented pathway activation, and orange represented pathway inhibition.

Pseudo-time and trajectory analysis suggested that all cells were distributed in one root and in two main branches (branches I and II). The results revealed that clusters 0, 3, 4, and 5 were mainly located in the roots, containing T and B cells; clusters 1, 2, and 6 were mainly located in branch I, containing macrophages and monocytes; and clusters 7, 8, and 9 were mainly located in branch II, containing cancer cells, cancer stem cells, and some B cells (Figure 2A). In addition, this work identified 240 LDRGs in branch I and 213 LDRGs in branch II (Supplementary Tables S1, S2). To understand the correlation between the molecular functions of LDRGs and the different differentiation trajectories, we used the WebGestalt online tool to perform GSEA. The LDRGs in branch I were dramatically associated with repressed immune-related pathway response and active ribosome activity, whereas the LDRGs in branch II were related with vibrant immune-related pathways and susceptibility to bacterial or viral infections. Collectively, these findings demonstrated that LUAD cells in different differentiated directions exhibited different biological tumor characteristics (Figures 2B,C).

### Correlation Between Lung Adenocarcinoma Subtypes Based on Lung Adenocarcinoma Differentiation-Related Genes and Different Overall Survival and Clinical Characteristics

**FIGURE 3:**
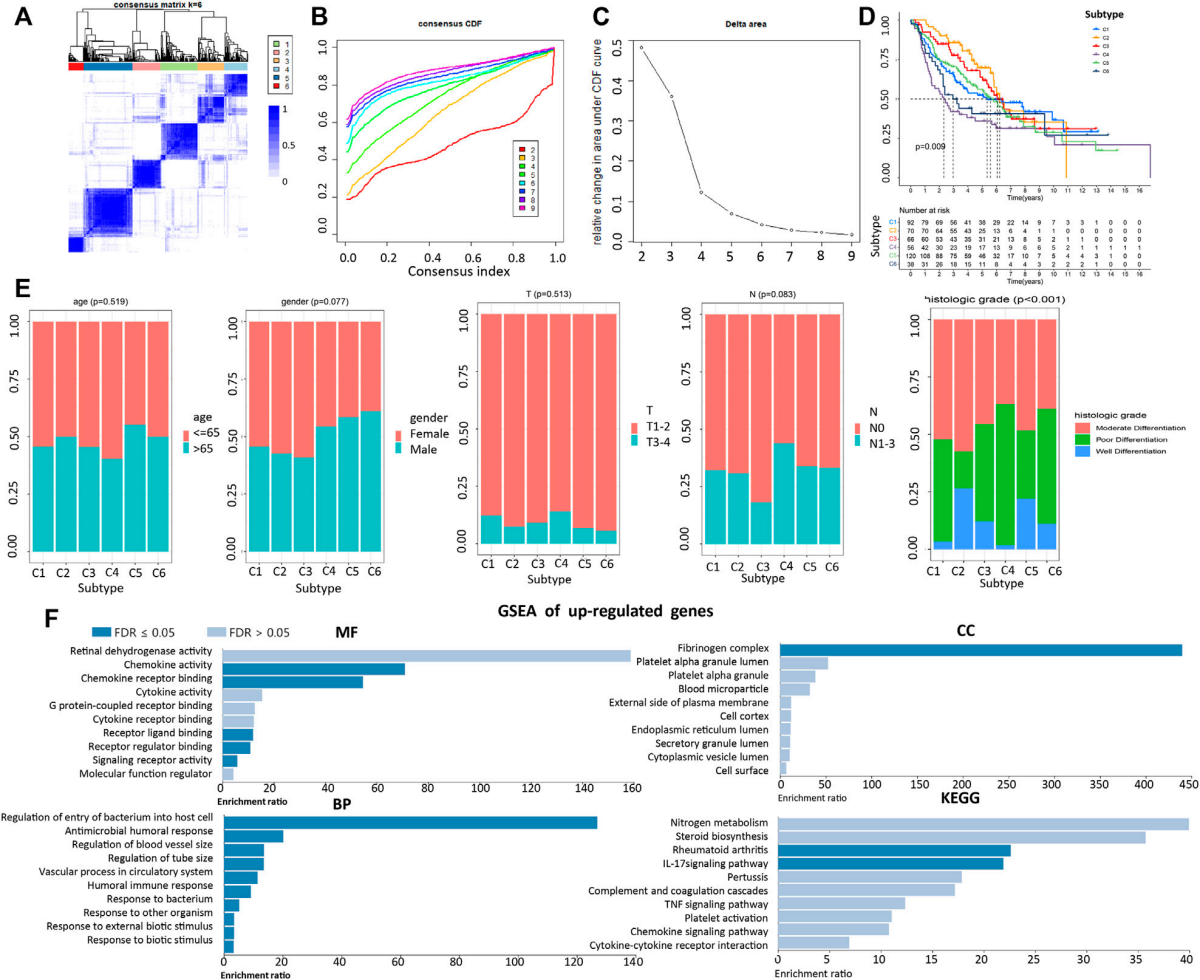
Correlation of LUAD subtypes based on LDRGs with different OS and clinical characteristics. **(A)** Consensus clustering matrix of GEO cohort (k = 6). **(B,C)** CDF curve of consensus score (k = 2–9) in GEO cohort. **(D)** Kaplan-Meier survival analysis of the 6 subtypes of LUAD patients in the GEO cohort. **(E)** Comparisons of the clinicopathological variables with six subtypes patients. **(F)** MF, CC, BP, and KEGG analysis of co-upregulated genes. An FDR < 0.05 was considered statistically significant.

A LDRG-based consensus clustering analysis was performed on 443 LUAD patients in GSE68465, and all acquired samples were classified into six subtypes (*k* value = 6, Figures 3A–C). Kaplan-Meier survival analysis revealed that subtype 2 (C2) had the highest overall survival (OS), whereas subtype 4 (C4) had the worst OS (log-rank *p* = 9 × 10^–3^, Figure 3D). In addition, we compared the clinicopathological characteristics of LUAD among different subtypes of patients in the GSE68465 cohort. As shown in Figure 3E, there were significant differences among the six subtypes pertaining to histologic grades. However, no significant differences were observed in other variables among all subtypes.

To compare genes related to differential histologic grades in different subtypes, with an adjusted *p* value < 0.05 and fold change > 2 set as parameters, the EdgeR was conducted to analyze differences between C4 and C2 and between C6 and C2. Taking the intersection, the results showed that there were 18 co-upregulated and 14 co-downregulated genes. GSEA indicated that upregulating genes were closely related to the increased sensitivity of cells to external stimuli, to decreased cell resistance to bacterial infection, and to the widening of the blood vessel diameter (Figure 3F). In contrast, enrichment results pertaining to downregulated genes were not significant (Supplementary Figure S3).

### Analysis of Tumor Microenvironment Scores, Prognostic Analysis in Immune Cell Infiltration, and Immune Checkpoint Genes

**FIGURE 4:**
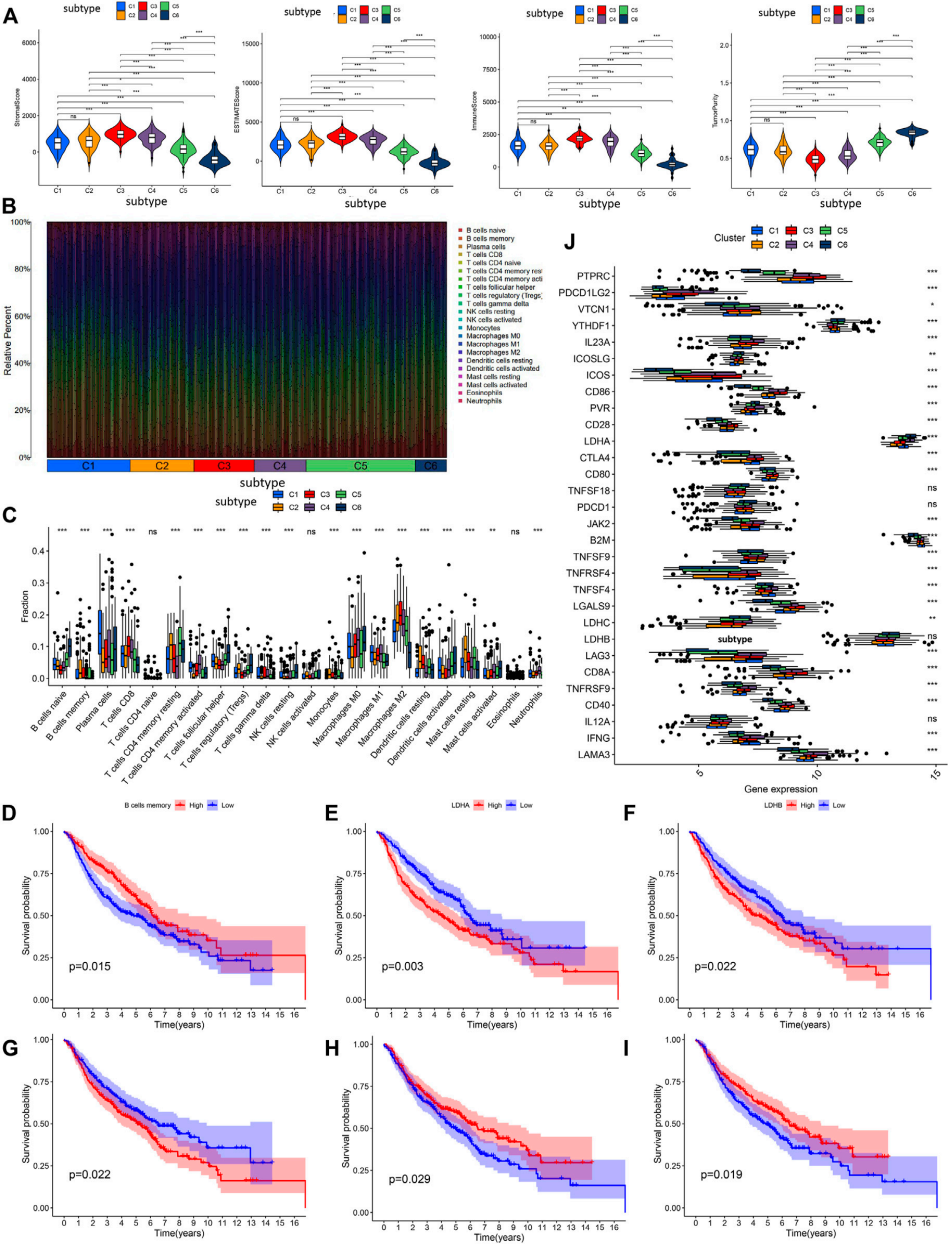
The analysis of tumor microenvironment scores, and prognostic analysis in immune cell infiltration and immune checkpoint genes. **(A)** Difference analysis of immune cell score, stromal cell score, ESTIMATE score and tumor purity among the six subtypes. **(B)** The accumulation percentage map of 22 kinds of immune cells in six subtypes from different LUAD samples. **(C)** Box plots of infiltration density of 22 kinds of immune cells in different subtypes. Kaplan-Meier survival analysis of immune cells **(D)** and immune checkpoints **(E–I)**. **(J)** Differential expression analysis of 30 recognized immune checkpoints in different subtypes. ns *p* > 0.05, *p < 0.05, **p < 0.01, ***p < 0.001.

Based on tumor microenvironment scores, our results revealed that stromal/ESTIMATE/immune scores in C3, C4, C5, and C6 decreased, whereas tumor purity was gradually increased in C3, C4, C5, and C6 (Figure 4A). The content of the 22 immune cells in each LUAD sample was calculated by the CIBERSORT algorithm and was visualized through an accumulation percentage map, where different colors represent different immune cells (Figure 4B). Afterward, the Wilcoxon test was used to compare the infiltration density of the immune cells in different subtypes (Figure 4C), and the results found that the immune cells were significantly different among the six subtypes examined (*p* < 0.01), except for the activated CD4 T cells, activated NK cells, and eosinophils. The Kaplan-Meier survival analysis showed that the infiltration density of memory B cells was positively correlated with patient survival (*p* = 0.015, Figure 4D). Memory B cell infiltration density was found to be the highest in C2, which translates to improved OS, whereas memory B cell infiltration density was the lowest in C6, which means poor OS.

Expression of the 30 immune checkpoints among LUAD subtypes was measured and compared (Figure 4J), and our results found that immune checkpoints were significantly different among the six subtypes (*p* < 0.05), except for interleukin 12A (IL12A), lactate dehydrogenase B (LDHB), programmed cell death 1 (PDCD1), and TNF superfamily member 18 (TNFSF18). It is worth noting that downregulation of LDHB predicted a better OS (Figure 4F). LDHA was downregulated in C2, and the Kaplan-Meier analysis indicated that lowly expressed LDHA predicted better OS (Figure 4E). CD28 and TNFRSF4 were upregulated in C3, and the Kaplan-Meier analysis indicated that highly expressed CD28 and TNFRSF4 predicted better OS (Figures 4H,I). Furthermore, JAK2 was upregulated in C4, and the Kaplan-Meier analysis indicated that highly expressed JAK2 predicted worse OS (Figure 4G). These results provide a molecular basis for understating that the different subtypes have different OS.

### Construction of a Prognostic Risk Scoring Model to Predict Lung Adenocarcinoma Patient Survival

Based on previous studies, 313 genes, consisting of 291 LDRGs, 18 significantly upregulated genes related to histologic grade and four immune checkpoint genes were further screened by univariate analysis, LASSO, and multivariate Cox analysis (Supplementary Tables S3, S4). Ultimately, 14 genes were generated in the TCGA training cohort. According to the 95% confidence interval of these genes, we finally identified eight significant survival-predicting genes: c-type lectin domain containing 7A (CLEC7A, HR = 0.69, *p* < 0.001), baculoviral IAP repeat containing 3 (BIRC3, HR = 1.19, *p* = 0.032), superoxide dismutase 1 (SOD1, HR = 1.62, *p* = 0.007), versican (VCAN, HR = 1.17, *p* = 0.039), kynureninase (KYNU, HR = 1.14, *p* = 0.032), CC motif chemokine ligand 20 (CCL20, HR = 1.10, *p* = 0.004), cancer/testis antigen 1B (CTAG1B, HR = 2.40, *p* < 0.001), and CD69 molecule (CD69, HR = 0.82, *p* = 0.022). Furthermore, the Supplementary Figure S4 demonstrates the expression levels of eight prognostic genes in cell profiles. It is shown that CLEC7A, VCAN, and KYNU were significantly upregulated in monocytes, BIRC3 and SOD1 were mainly distributed in T cells, CCL20 was highly expressed in tumor cells, and CD69 was highly expressed in B cells. CTAG1B was filtered out during quality control.

The prognostic risk scoring model was developed based on the above eight prognostic-related genes, and the formula was as follows:

Risk score = Exp_CLEC7A_ × (−0.377) + Exp_BIRC3_ × 0.170 + Exp_SOD1_ × 0.483 + Exp_VCAN_ × 0.156 + Exp_KYNU_ × 0.132 + Exp_CCL20_ × 0.099 + Exp_CTAG1B_ × 0.877 + Exp_CD69_ × (−0.201).

**FIGURE 5:**
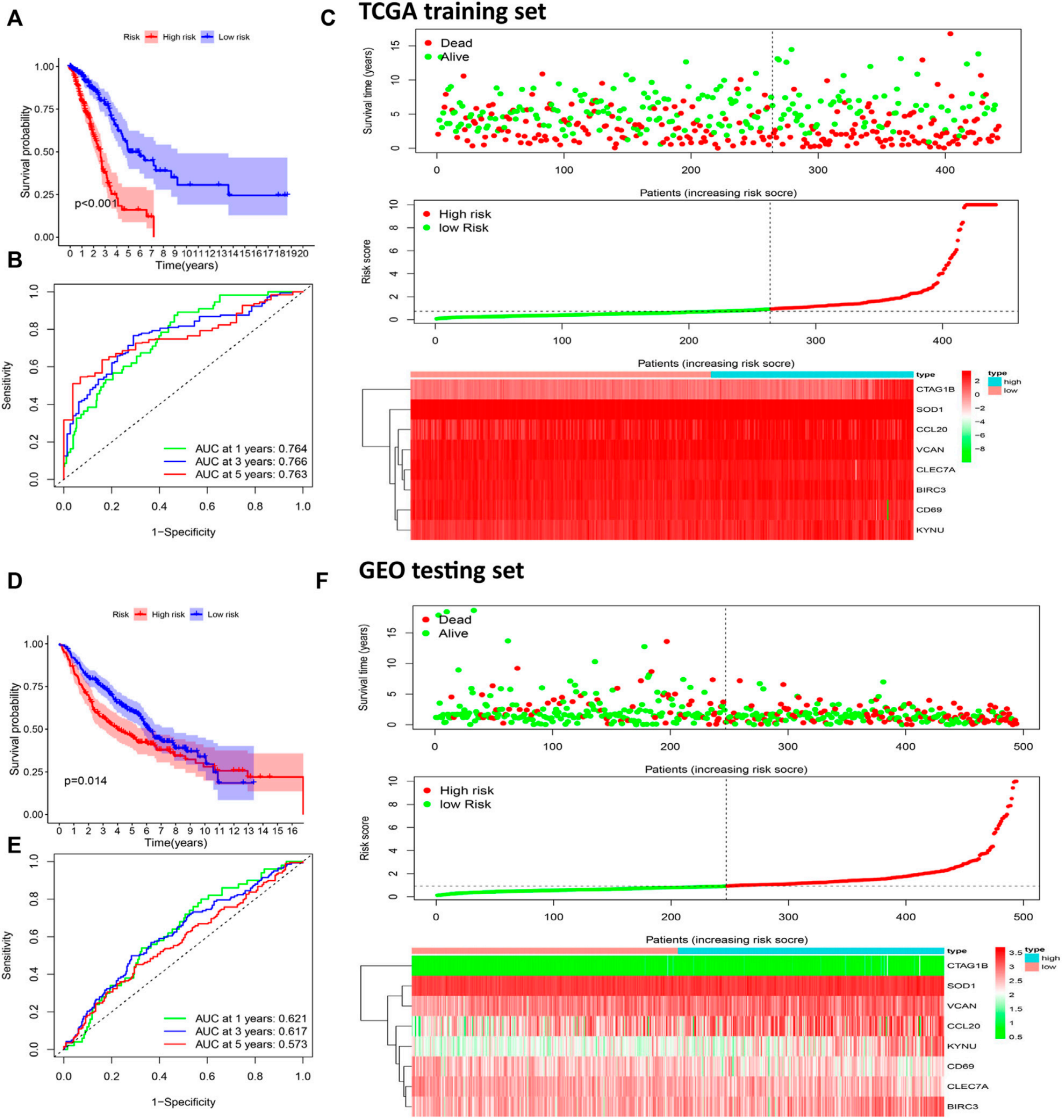
Construction of a prognostic risk scoring model to predict LUAD patient survival. Kaplan-Meier survival analysis was performed to calculate the OS of high-risk and low-risk groups patients in the TCGA training set **(A)** and GEO test set **(D)**. The ROC curve was used to verify the ability of the risk model to judge the 1-, 3-, and 5-year OS rates of patients in the TCGA **(B)** and GEO set **(E)**. Risk score analysis of eight prognostic-related genes in TCGA **(C)** and CGGA **(F)** sets. Upper: The survival status and time of LUAD patients distributed by risk score. Middle: The patients were classified into a high-risk group or a low-risk group by using the median value of the risk score. Bottom: Heat maps of the expression levels of eight prognostic-related genes.

According to this formula, the risk scores of all patients in the TCGA training set and the GEO testing set were calculated, and the patients were divided into high- and low-risk groups based on the median of the risk scores (Figure 5C). The Kaplan-Meier survival analysis showed that OS of patients in the high-risk group was significantly poorer than the OS in the low-risk group both in the training set (*p* < 0.001, Figure 5A) and the testing set (*p* = 0.014, Figure 5D). The areas under the receiver operating characteristic curves (ROC) for predicting 1-, 3-, and 5-year OS were 0.764, 0.766, and 0.760, respectively, in the training set (Figure 5B) and 0.621, 0.617, and 0.573, respectively, in the testing set (Figure 5E). These results showed that this prognostic risk score model could be used as a reliable prognostic predictor for LUAD patients.

### The Key Role of CCL20 in Lung Adenocarcinoma

**FIGURE 6:**
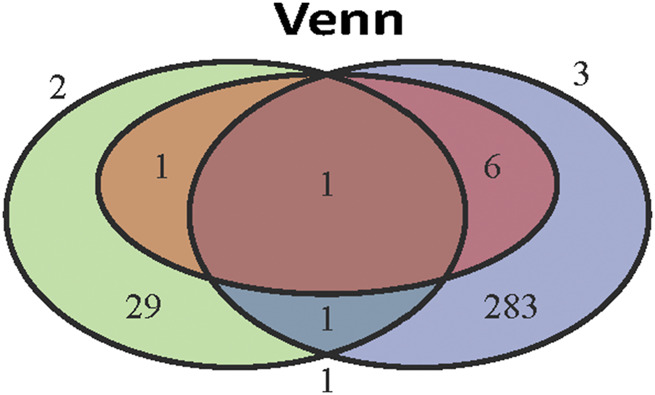
The key role of CCL20 in LUAD. Venn diagram of eight independent risk scoring genes, 32 genes related to poor differentiation and 291 LDRGs.

The intersection of 291 LDRGs and 32 genes related to histologic grade showed that they both had CCL20 and C-X-C motif chemokine ligand 8 (CXCL8). Furthermore, CCL20 was also included in the survival-predicting genes (Figure 6) and was mainly expressed in cancer cells of branch II.

### Establishment and Validation of a Nomogram for Predicting Patient OS Based on the The Cancer Genome Atlas Cohort

**FIGURE 7:**
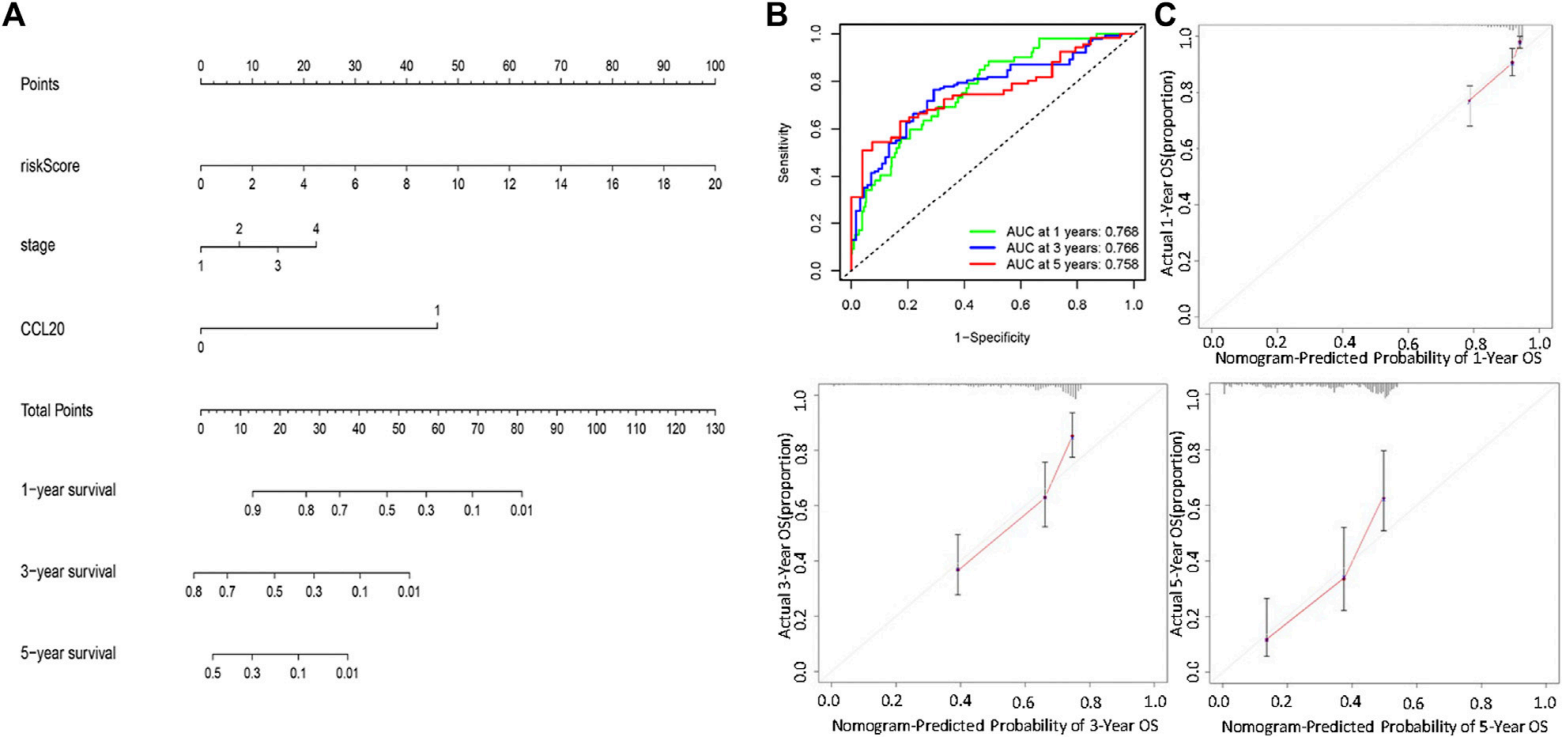
Establishment and validation of a nomogram for predicting patient OS based on the TCGA cohort. **(A)** Nomogram model to predict the prognosis of LUAD patients based on the TCGA cohort stage, CCL20 mutation situation and risk score were included in the prediction model. **(B)** The 1-year, 3-year, and 5-year ROC curve were used to assessed the prognostic performance of the nomogram. **(C)** The calibration curves for predicting the 1-year, 3-year, and 5-year OS.

Univariate and multivariate Cox regression analysis showed that stage, risk score, and CCL20 mutation status could be used as independent factors for the prognosis of LUAD (Supplementary Tables S5, S6). Patients with higher risk scores and later stage had poorer prognosis. CCL20 mutations were also associated with poor prognosis. Finally, a prognostic nomogram was successfully developed to predict the OS rate of LUAD patients based on the TCGA cohort (Figure 7A). Time-dependent ROC analysis showed that the nomogram could effectively predict the 1-, 3-, and 5-year OS rates of patients, with AUC values of 0.768, 0.766, and 0.758, respectively (Figure 7B). The calibration chart also revealed that the predicted OS rate was consistent with the actual observations in the TCGA cohort (Figure 7C).

## Discussion

Although the incidence of LUAD is consistently reduced year by year, as a result of public education and implementation of etiological preventive measures, such as quitting smoking, it still remains a global public health issue due to its respective high mortality rates (Siegel et al., 2021). To date, there is a lack of effective prediction models that could be used to guide clinical prognosis evident. Most tumors originate from a single cell, and tumor progression is caused by the gradual selection of more aggressive and more resistant dominant cancer cell variant sublines due to acquired genetic instability (Nowell, 1976). The strong drug resistance caused by heterogeneity greatly increases the complexity of tumors and reflects the increasing difficulty of drug design, thus rendering the prognosis of cancer patients unsatisfactory (Fedele et al., 2014). LUAD is the most common respiratory tumor with extremely high heterogeneity. In this study, we analyzed the factors affecting LUAD heterogeneity based on the RNA-seq and somatic mutation data from TCGA and the scRNA-seq and RNA-seq data from GEO. Single-cell trajectory analysis verified LUAD heterogeneity, and the subsequent enrichment analysis exhibited that this heterogeneity was closely related to the activation and suppression of the immune system function (Satija et al., 2015; Lall et al., 2018). Eventually, CLEC7A, CD69, SOD1, BIRC3, VCAN, KYNU, CCL20, and CTAG1B were identified as the eight most significant, independent predictive genes. Based on these eight genes, we established a risk scoring model and combined the tumor stage and the CCL20 mutation status to successfully develop a 1-, 3-, and 5-year prognostic nomogram. With high predictive power, the nomogram could provide certain evidence support for clinicians to predict the possibility of patient survival time, thus enabling clinicians to formulate individualized optimal treatment plans.

CLEC7A, also known as Dectin-1 and CD369, is a pattern recognition receptor expressed by myeloid phagocytes (macrophages, dendritic cells, and neutrophils) and plays a pivotal role in antifungal response, antimicrobial reaction, and autoimmunity through phagocytosis, respiratory burst, cytokines, and others (Goodridge et al., 2011; Mattiola et al., 2019). Besides, accumulated evidence has suggested that CLEC7A had dual effects on cancer, namely, inhibiting and promoting cancer (Tone et al., 2019). CLEC7A has been widely studied in pancreatic cancer, kidney cancer, and liver cancer, etc., but the true role of this gene remains controversial (Seifert et al., 2015; Xia et al., 2016; Daley et al., 2017). CLEC7A, which is highly expressed in M2-like tumor-associated macrophages, is linked to galectin 9, thus leading to tolerogenic macrophage programming and adaptive immunosuppression that contribute to tumor exacerbation (Daley et al., 2017; Zhou et al., 2021a). Conversely, activation of CLEC7A can exert effective anti-tumor immunity by enhancing the expression of interleukin-33 and interleukin-9 in dendritic cells and by inducing differentiation of naive CD4^+^T cells into T-helper 9 cells (Zhao et al., 2016; Chen et al., 2018). CLEC7A could also stimulate the secretion of cytokines, such as interleukin-13, interleukin-1, and TNF-α in macrophages to activate macrophage tumoricidal response (Deng et al., 2018; Alaeddine et al., 2019). This opposite effect may be caused by the activation of different types of immune cells by CLEC7A in different pathways. So far, little is known regarding the relationship between CLEC7A and lung cancer. Chiba et al. found that CLEC7A could promote natural killer cells to secrete cytotoxic factors and thus fight lung cancer through cell-to-cell contact (Chiba et al., 2014). In this study, CLEC7A had a positive effect on the prognosis of LUAD patients, which was consistent with the findings of previous studies (Zou et al., 2020; Zhou et al., 2021b; Lin et al., 2021).

In addition, the key role of CCL20 in LUAD was identified in this study. CCL20, also called liver and activation-regulated chemokine, macrophage inflammatory protein 3 alpha, and small-inducible cytokine A20, is highly expressed in many types of cancer, including NSCLC, ovarian cancer, colorectal cancer, penile cancer, breast cancer, gastric cancer, and pancreatic cancer (Han et al., 2015; Liu et al., 2016; Lee et al., 2017; Wang et al., 2019b; Liu et al., 2020a; Mo et al., 2020). This ligand has been found to enhance the migration and proliferation of cancer cells through the CCL20/CCR6 signaling pathway and to inhibit cell apoptosis, which directly promotes cancer progression. In addition, CCL20 can indirectly promote cancer progression through immune cell control to reshape tumor microenvironment. For instance, Wei et al. concluded that the high expression of CCL20 in LUAD could enhance CCL20/CCR6/ERK signal transduction, thereby promoting the proliferation and migration of tumor cells (Wei et al., 2019). Furthermore, Wang et al. believed that NSCLC cells produce CCL20 in an autocrine and paracrine manner. Overexpression of CCL20 overactivates the MAPK-PI3K signaling pathway, thus leading to high chemoresistance, accelerated proliferation, or a more stable ability to reside in new locations of cancer cells (Wang et al., 2016). In this work, CCL20 was found to be highly expressed mainly in branch II and concentrated in tumor cells. Enrichment analysis showed that the significant CCL20 upregulation can increase the sensitivity of tumors to extracellular stimuli and promote bacterial invasion of host cells, leading to instability of the intracellular environment, but it can also increase the diameter of blood vessels in the tumor environment to meet the needs of tumor proliferation and migration. Furthermore, our findings showed that CCL20 was significantly negatively correlated with the patient’s OS. Consistent with previous research, CCL20 was confirmed to be an important gene involved in the evolution of LUAD tumors, poor histologic grade, and independent prognosis of patients in our work.

CD69 upregulation could induce LUAD cell apoptosis and inhibit tumor proliferation, invasion, and metastasis, thus suggesting a good prognosis. BIRC3, VCAN, CTAG1B, SOD1, and KYNU promoted the survival and proliferation of cancer cells, inhibited cancer cell apoptosis, and were related to poor prognosis and treatment resistance (Jin et al., 2018; Liu et al., 2020b; Al‐Mansoob et al., 2021; Frazzi, 2021; Li et al., 2021). In this work, these genes could also be used as potential prognostic genes for LUAD, and their impact on LUAD was consistent with the findings of previous reports.

Tumor immunotherapy has proven to be very effective because it can manipulate the individual’s immune system to identify and attack cancer cells, thereby inducing a long-lasting and strong anti-tumor response (Gotwals et al., 2017; Kennedy and Salama, 2020). Immunotherapy has achieved amazing effects in solid tumors, such as melanoma, bladder cancer, and breast cancer, but the effective survival benefit of lung cancer has only recently been confirmed (van den Bulk et al., 2018). However, it should not be ignored that immunotherapy is not applicable to all patients with LUAD (Zhao et al., 2019; Hegde and Chen, 2020). Unfortunately, some patients have poor sensitivity to immunotherapy, but also treatment side effects, such as pneumonia, can further damage the already poor lung reserve, leading to accelerated disease deterioration (Steven et al., 2016). Therefore, it is very important to distinguish patients who are sensitive to immunotherapy. The tumor immune dysfunction and elimination (TIDE) algorithm came into being. The results of this study showed that the TIDE score of low-risk patients was significantly higher than that of high-risk patients, indicating that high-risk patients are bound to benefit more from immunotherapy and thus get a better prognosis compared to low-risk patients (Supplementary Figure S5). In general, a risk scoring model composed of eight independent genes could provide a preliminary estimate of patients’ responsiveness to immunotherapy, thereby minimizing the negative effects of drug toxicity.

Our research also has certain limitations. In this research, we only analyzed the molecular phenotypes and existing clinical data that we were interested in. Phenotypes such as gene methylation, gene mutations, long non-coding RNA, competing endogenous RNA, and clinicopathological characteristics, such as smoking history, chemotherapy and radiotherapy history, history of underlying diseases, history of disease progression, etc., were not considered due to the lack of unified standard data. Consequently, survival prediction models necessitate continuous improvement.

## Conclusion

The current research analyzed the genes that affected tumor heterogeneity and established a risk scoring model consisting of eight independent prognostic genes through data dimensionality reduction. The nomogram that combined risk scores, clinicopathological variables, and CCL20 mutation status provided a visual and accurate method for predicting the 1-, 3-, and 5-year survival rates of patients. In conclusion, this study emphasizes the impact of tumor heterogeneity on predicting patient OS and immunotherapy response and underlines a potential therapeutic target for the treatment of LUAD.

## Data Availability

The datasets presented in this study can be found in online repositories. The names of the repository/repositories and accession number(s) can be found in the article/Supplementary Material.

## Glossary

BIRC3: Baculoviral IAP repeat containing 3
BP: Biological process
CC: Component
CCL20: CC motif chemokine ligand 20
CD69: CD69 molecule
CDF: The cumulative distribution function
CLEC7A: c-type lectin domain containing 7A
CTAG1B: Cancer/testis antigen 1B
GEO: Gene Expression Omnibus
GO: Geneontology
ICG: Immune checkpoint gene
KEGG: Kyoto encyclopedia of genes and genomes
KYNU: Kynureninase
LASSO: Least absolute shrinkage selection operator
LDRGs: LUAD differentiation-related genes
LUAD: Lung adenocarcinoma
LUSC: Lung squamous cell carcinoma
MF: Molecular function
NSCLC: Non-small cell lung cancer
OS: Overall survival
PC: Principal components
PCA: Principal component analysis
ROC: Receiver operating characteristic curves
SCLC: Small cell lung cancer
SOD1: Superoxide dismutase one
TCGA: The Cancer Genome Atlas
TF: Transcription factors
TIDE: The tumor immune dysfunction and elimination
t-SNE: The t-distributed stochastic neighbor embedding
VCAN: Versican
