# Supplementary material for: Establishment of a Prognostic Model of Lung Adenocarcinoma Based on Tumor Heterogeneity
Source: Front Mol Biosci. 2022 Apr 11;9:807497. doi: 10.3389/fmolb.2022.807497 (PMC9035852; doi:10.3389/fmolb.2022.807497)
Supplement: Supplementary file 1 [file DataSheet1.docx]

Supplementary Material

**
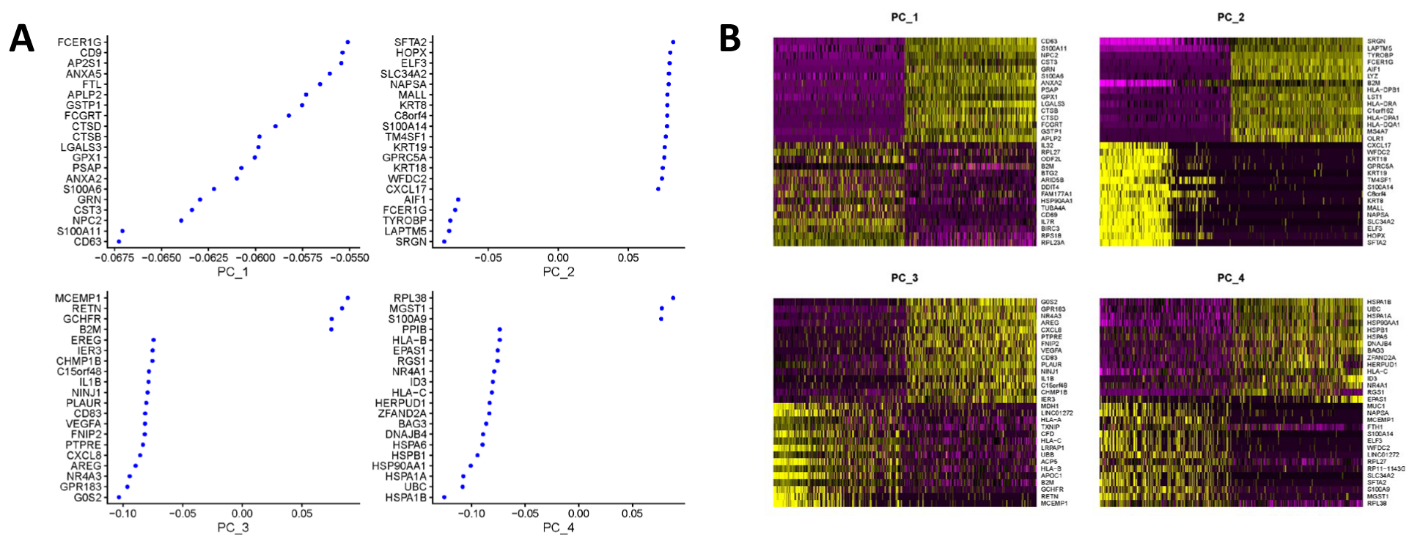
 Supplementary Figure 1. Significantly related genes in PC1 to PC4.** (A) The dot plot showed the top 20 significantly related genes in each PC. The X axis represented the correlation coefficient. (B) The heatmaps showed the expression patterns of the top 20 significantly related genes in each PC. The colors from purple to yellow indicated the gene expression level from low to high.

**
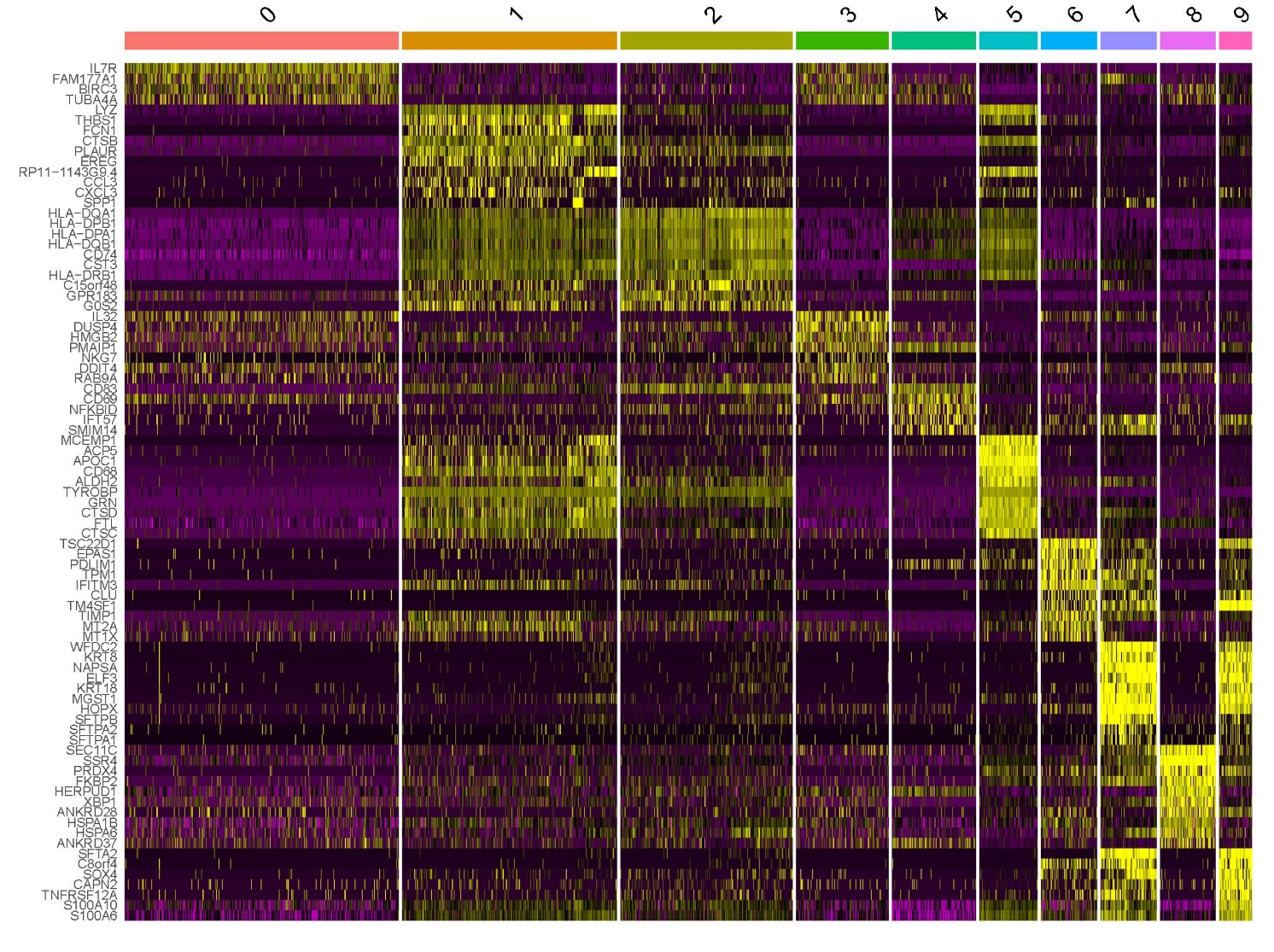
**

**Supplementary Figure 2. Heat map of the expression of marker genes in 10 clusters.** Yellow represented high expression and purple represents low expression.

**
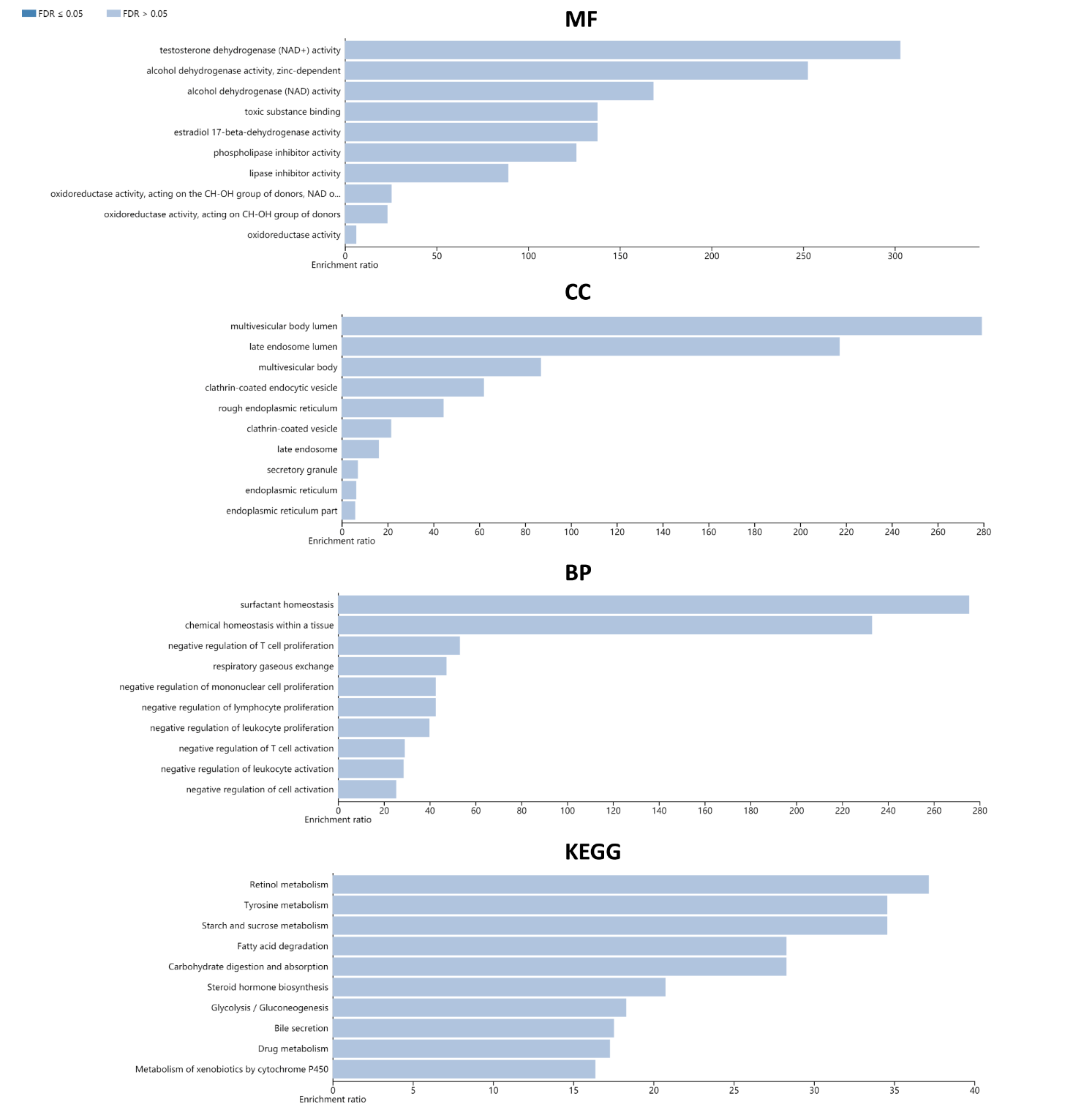
**

**Supplementary Figure 3. MF, CC, BP, KEGG analysis of co-downregulated genes.** Take FDR<0.05 as the significant parameter. The results showed that the enrichment of down-regulated genes is not significant.

**
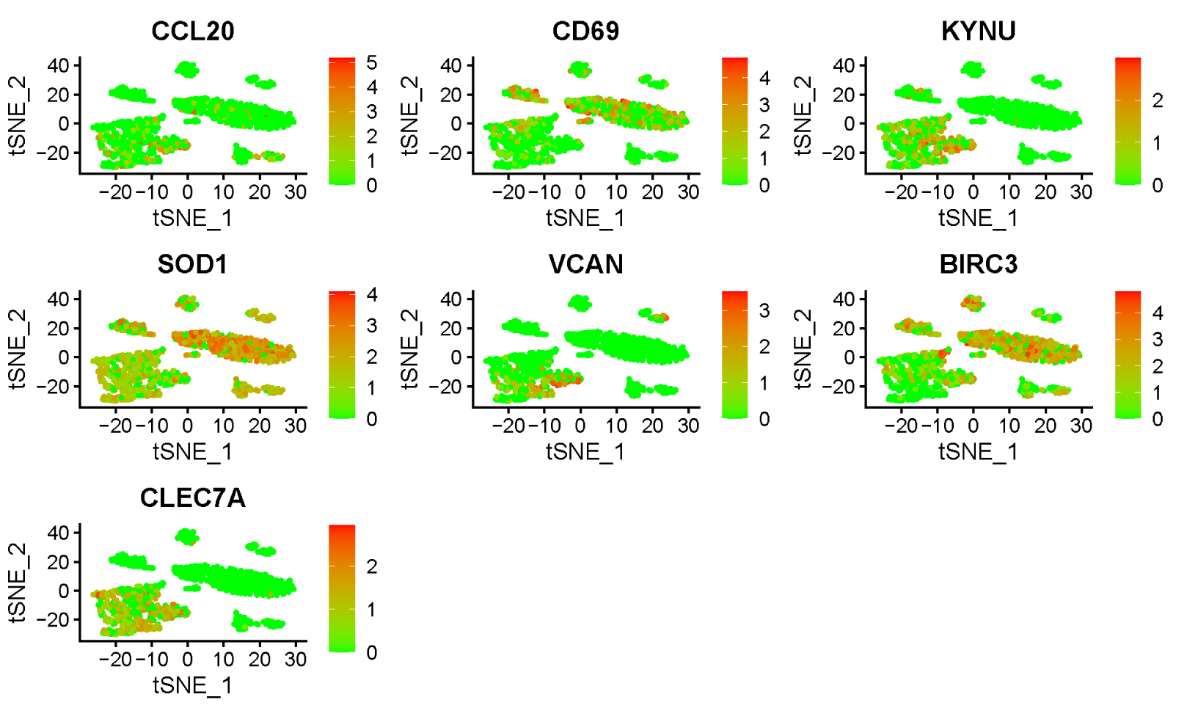
**

**Supplementary Figure 4. The expression levels of 8 risk genes in 6 types of cells.** Red indicated high expression and green indicated low expression.

**
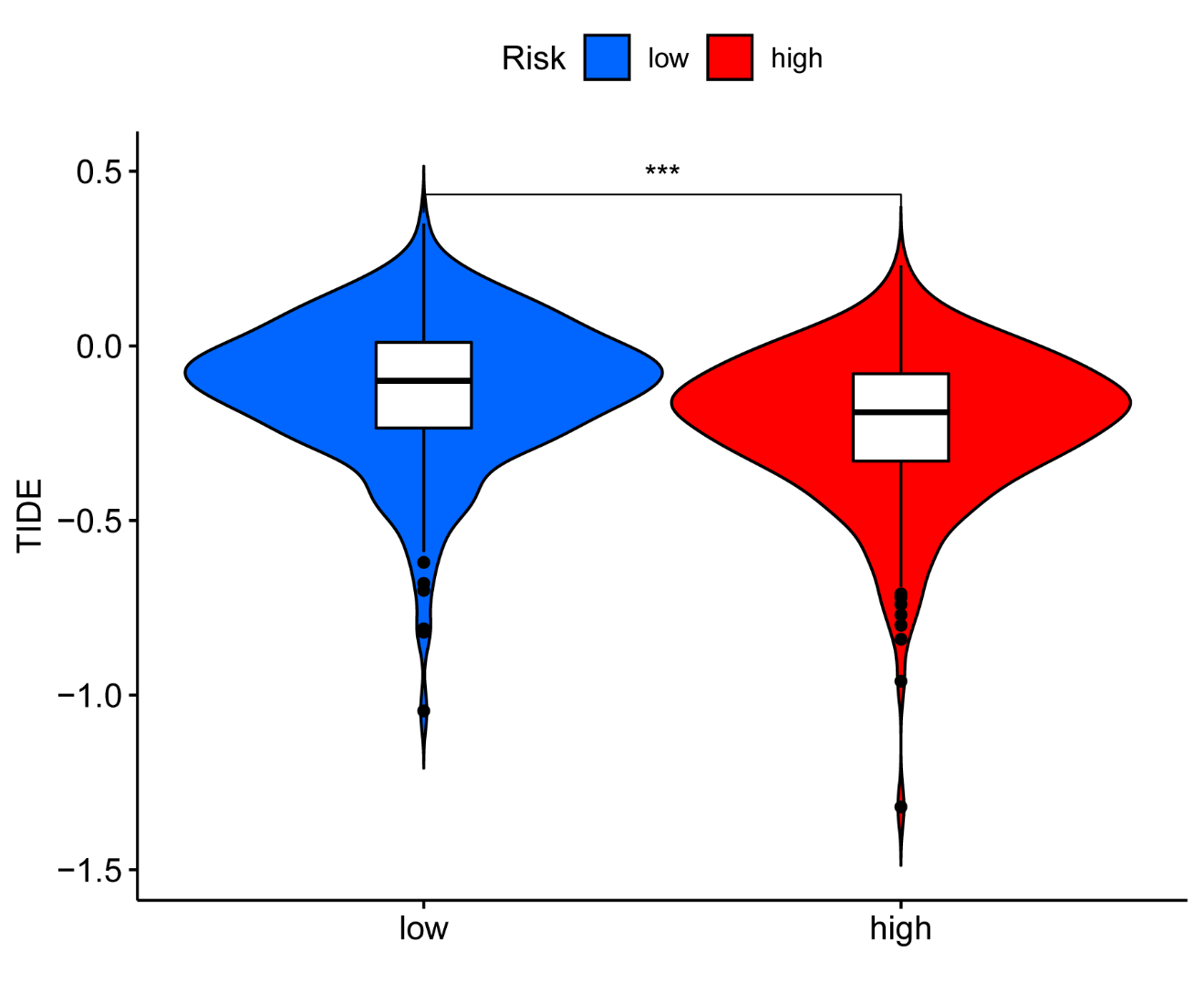
**

**Supplementary Figure 5. Analysis of differences in TIDE scores of high- and low-risk patients.** The results showed that the TIDE score of low-risk patients was significantly higher than that of high-risk patients.

**Supplementary Table 1. Variance analysis results of 240 genes in the branch I based on sc-RNA seq.**

| Gene | P value | log_2_FC | P value-adjusted |
| --- | --- | --- | --- |
| CST3 | 3.90E-225 | -3.86091 | 1.23E-221 |
| NPC2 | 7.00E-213 | -2.65307 | 2.21E-209 |
| RPL13A | 5.62E-190 | 1.02734 | 1.78E-186 |
| RPL21 | 1.06E-189 | 1.13237 | 3.37E-186 |
| RPS26 | 1.02E-188 | 1.144582 | 3.23E-185 |
| RPS27A | 3.05E-185 | 1.09665 | 9.64E-182 |
| GRN | 4.33E-185 | -2.82218 | 1.37E-181 |
| CD63 | 2.40E-182 | -2.42965 | 7.60E-179 |
| GPX1 | 5.53E-181 | -2.50903 | 1.75E-177 |
| RPS3 | 2.68E-177 | 1.074073 | 8.48E-174 |
| FTL | 4.07E-176 | -3.29356 | 1.29E-172 |
| RPS27 | 8.38E-176 | 1.035262 | 2.65E-172 |
| GSTP1 | 1.09E-172 | -2.17456 | 3.44E-169 |
| FCGRT | 3.25E-168 | -2.10985 | 1.03E-164 |
| S100A11 | 1.01E-164 | -2.36666 | 3.21E-161 |
| PSAP | 1.86E-164 | -2.62422 | 5.88E-161 |
| S100A6 | 8.65E-163 | -2.40712 | 2.73E-159 |
| IFITM3 | 9.71E-159 | -2.91368 | 3.07E-155 |
| CTSB | 4.94E-153 | -2.57947 | 1.56E-149 |
| AIF1 | 6.86E-151 | -2.80424 | 2.17E-147 |
| ALDH2 | 2.27E-147 | -2.07115 | 7.16E-144 |
| TYROBP | 5.44E-147 | -3.77684 | 1.72E-143 |
| NEAT1 | 7.54E-147 | -1.98268 | 2.38E-143 |
| LYZ | 8.90E-144 | -5.43877 | 2.81E-140 |
| FCER1G | 2.50E-142 | -3.05092 | 7.90E-139 |
| FCGR2A | 3.22E-139 | -2.15741 | 1.02E-135 |
| CXCR4 | 2.70E-138 | 1.901578 | 8.55E-135 |
| HLA-DPA1 | 6.12E-138 | -3.18126 | 1.93E-134 |
| HLA-DRB1 | 2.27E-137 | -3.19514 | 7.18E-134 |
| PLAUR | 1.51E-136 | -2.48939 | 4.77E-133 |
| HLA-DMA | 1.50E-135 | -1.95812 | 4.73E-132 |
| CTSH | 3.83E-135 | -1.78248 | 1.21E-131 |
| LST1 | 1.89E-134 | -2.33852 | 5.99E-131 |
| ANXA2 | 2.48E-133 | -1.90814 | 7.84E-130 |
| RPSA | 4.90E-132 | 1.054343 | 1.55E-128 |
| CD74 | 2.03E-130 | -2.59932 | 6.42E-127 |
| CD68 | 5.15E-130 | -2.61026 | 1.63E-126 |
| ANXA5 | 7.15E-130 | -1.58865 | 2.26E-126 |
| GSN | 2.12E-128 | -1.74219 | 6.70E-125 |
| AP2S1 | 1.23E-127 | -1.48729 | 3.88E-124 |
| HLA-DRA | 2.88E-127 | -3.01327 | 9.11E-124 |
| SERF2 | 6.81E-127 | -1.11746 | 2.15E-123 |
| SERPINA1 | 1.28E-124 | -2.08482 | 4.06E-121 |
| TIMP1 | 1.37E-124 | -2.84499 | 4.34E-121 |
| RAB31 | 1.69E-124 | -1.74764 | 5.35E-121 |
| MS4A6A | 7.39E-123 | -2.39836 | 2.34E-119 |
| ACTB | 4.71E-121 | -1.57256 | 1.49E-117 |
| MS4A7 | 1.18E-119 | -2.19256 | 3.74E-116 |
| TMSB10 | 2.57E-119 | -1.26895 | 8.11E-116 |
| CSTA | 1.86E-118 | -1.97554 | 5.88E-115 |
| IER3 | 2.04E-118 | -2.21682 | 6.46E-115 |
| TGFBI | 8.22E-117 | -1.64374 | 2.60E-113 |
| HLA-DQB1 | 1.50E-116 | -2.52436 | 4.73E-113 |
| OLR1 | 2.42E-116 | -2.21065 | 7.66E-113 |
| LGALS3 | 2.83E-116 | -2.07472 | 8.94E-113 |
| CD9 | 6.97E-116 | -1.79357 | 2.20E-112 |
| CXCL16 | 2.77E-115 | -1.59382 | 8.75E-112 |
| CTSD | 4.46E-115 | -2.97437 | 1.41E-111 |
| APLP2 | 1.03E-113 | -1.47532 | 3.26E-110 |
| TSC22D3 | 1.36E-113 | 1.611986 | 4.30E-110 |
| HLA-DMB | 1.80E-112 | -1.8208 | 5.68E-109 |
| SAT1 | 7.18E-112 | -1.53177 | 2.27E-108 |
| C1orf162 | 6.89E-111 | -1.96846 | 2.18E-107 |
| SARAF | 2.07E-110 | 1.505231 | 6.55E-107 |
| GLUL | 1.50E-109 | -1.94786 | 4.74E-106 |
| ASAH1 | 2.29E-109 | -1.53333 | 7.25E-106 |
| HLA-DPB1 | 4.61E-108 | -2.76244 | 1.46E-104 |
| VAMP8 | 1.23E-107 | -1.51621 | 3.90E-104 |
| LGALS1 | 1.96E-107 | -1.73381 | 6.21E-104 |
| COMT | 2.78E-106 | -1.12897 | 8.79E-103 |
| KLF4 | 5.65E-104 | -1.46751 | 1.79E-100 |
| H2AFY | 1.00E-102 | -1.28838 | 3.17E-99 |
| ATOX1 | 4.20E-102 | -1.16182 | 1.33E-98 |
| PPT1 | 4.46E-102 | -1.36876 | 1.41E-98 |
| CAPG | 1.34E-100 | -1.67778 | 4.23E-97 |
| ETS2 | 2.22E-100 | -1.13943 | 7.01E-97 |
| HLA-DQA1 | 6.04E-100 | -2.59268 | 1.91E-96 |
| ATP6V0B | 4.28E-98 | -1.2981 | 1.35E-94 |
| CTSZ | 7.27E-96 | -1.29101 | 2.30E-92 |
| CTSC | 1.70E-95 | -1.78971 | 5.37E-92 |
| S100A9 | 3.35E-95 | -2.44626 | 1.06E-91 |
| ATP6V1F | 6.65E-95 | -1.29701 | 2.10E-91 |
| FNIP2 | 4.66E-94 | -1.28592 | 1.47E-90 |
| CLEC7A | 7.38E-94 | -1.17528 | 2.33E-90 |
| TSPO | 1.43E-93 | -1.48006 | 4.51E-90 |
| TMSB4X | 1.55E-93 | -1.00476 | 4.90E-90 |
| RAB13 | 5.36E-93 | -1.10282 | 1.69E-89 |
| MGAT1 | 1.21E-92 | -1.28363 | 3.82E-89 |
| IL7R | 2.01E-91 | 2.440766 | 6.36E-88 |
| CTSS | 1.09E-90 | -1.60256 | 3.44E-87 |
| SPINT2 | 1.56E-89 | -1.26568 | 4.94E-86 |
| NCF2 | 2.59E-89 | -1.20781 | 8.19E-86 |
| GNG5 | 2.60E-89 | -1.10271 | 8.21E-86 |
| CSTB | 2.70E-89 | -1.68203 | 8.53E-86 |
| CXCL8 | 2.89E-89 | -3.30294 | 9.14E-86 |
| TXN | 3.21E-89 | -1.60471 | 1.02E-85 |
| HEXB | 4.56E-89 | -1.03599 | 1.44E-85 |
| LAMTOR2 | 8.18E-89 | -1.04165 | 2.59E-85 |
| CXCL2 | 9.68E-88 | -2.8043 | 3.06E-84 |
| PYCARD | 2.24E-86 | -1.13987 | 7.08E-83 |
| PLSCR1 | 4.42E-86 | -1.07859 | 1.40E-82 |
| SLC11A1 | 9.23E-85 | -1.59351 | 2.92E-81 |
| MNDA | 3.31E-84 | -1.33158 | 1.05E-80 |
| THBS1 | 6.09E-84 | -1.98234 | 1.93E-80 |
| BIRC3 | 1.69E-83 | 1.535866 | 5.33E-80 |
| BLVRB | 1.78E-83 | -1.03629 | 5.62E-80 |
| UPP1 | 2.18E-83 | -1.31125 | 6.89E-80 |
| RHOB | 3.22E-83 | -1.32155 | 1.02E-79 |
| SYNGR2 | 3.93E-83 | -1.0441 | 1.24E-79 |
| CEBPD | 9.12E-83 | -1.07537 | 2.88E-79 |
| CTSL | 9.76E-83 | -1.65716 | 3.09E-79 |
| CD81 | 6.58E-82 | -1.08594 | 2.08E-78 |
| ITM2B | 8.48E-82 | -1.28176 | 2.68E-78 |
| ACSL1 | 1.31E-81 | -1.10434 | 4.14E-78 |
| TREM1 | 1.81E-81 | -1.44101 | 5.72E-78 |
| S100A10 | 7.62E-81 | -1.46651 | 2.41E-77 |
| HSPA8 | 8.68E-81 | 1.056288 | 2.75E-77 |
| ATF3 | 1.03E-80 | -1.47087 | 3.27E-77 |
| MRC1 | 3.83E-80 | -1.55225 | 1.21E-76 |
| RNASE6 | 1.73E-79 | -1.32571 | 5.47E-76 |
| G0S2 | 2.78E-79 | -3.36713 | 8.79E-76 |
| PMP22 | 2.33E-78 | -1.33285 | 7.38E-75 |
| VIM | 4.49E-78 | -1.28541 | 1.42E-74 |
| MYL6 | 6.11E-78 | -1.0427 | 1.93E-74 |
| TCEB2 | 7.40E-78 | -1.16064 | 2.34E-74 |
| HBEGF | 6.65E-77 | -1.36989 | 2.10E-73 |
| APOC1 | 1.24E-76 | -3.48756 | 3.91E-73 |
| ARPC3 | 5.35E-76 | -1.00368 | 1.69E-72 |
| C5AR1 | 2.76E-75 | -1.34289 | 8.74E-72 |
| CD69 | 4.37E-75 | 2.196167 | 1.38E-71 |
| IGSF6 | 5.46E-75 | -1.1384 | 1.73E-71 |
| MYDGF | 8.50E-75 | -1.01044 | 2.69E-71 |
| CPVL | 8.60E-75 | -1.27149 | 2.72E-71 |
| LIMS1 | 9.49E-75 | -1.15929 | 3.00E-71 |
| MS4A4A | 3.28E-74 | -1.41261 | 1.04E-70 |
| PKM | 1.35E-73 | -1.07723 | 4.26E-70 |
| GPX4 | 1.71E-73 | -1.07564 | 5.40E-70 |
| SLC7A7 | 7.61E-73 | -1.0364 | 2.41E-69 |
| FBP1 | 7.99E-73 | -1.59349 | 2.53E-69 |
| PLEK | 1.77E-72 | -1.17907 | 5.58E-69 |
| ARPC1B | 2.05E-71 | -1.02183 | 6.47E-68 |
| PHACTR1 | 3.48E-71 | -1.17533 | 1.10E-67 |
| IFI6 | 6.76E-71 | -1.9633 | 2.14E-67 |
| C15orf48 | 1.43E-70 | -1.98969 | 4.53E-67 |
| CREM | 1.38E-69 | 1.316894 | 4.37E-66 |
| TKT | 1.42E-69 | -1.10429 | 4.49E-66 |
| IER5 | 3.94E-69 | -1.13661 | 1.25E-65 |
| CYBB | 1.35E-68 | -1.06823 | 4.26E-65 |
| RP11-1143G9.4 | 4.01E-68 | -2.17859 | 1.27E-64 |
| S100A13 | 8.09E-68 | -1.29364 | 2.56E-64 |
| GSTO1 | 1.54E-67 | -1.074 | 4.86E-64 |
| AVPI1 | 1.93E-67 | -1.02412 | 6.12E-64 |
| FKBP2 | 4.28E-67 | -1.00421 | 1.35E-63 |
| SLC16A10 | 3.89E-66 | -1.33345 | 1.23E-62 |
| CYBA | 8.61E-66 | -1.24207 | 2.72E-62 |
| ABL2 | 2.19E-65 | -1.12351 | 6.93E-62 |
| SGK1 | 5.37E-65 | -1.15802 | 1.70E-61 |
| ENO1 | 7.68E-65 | -1.02951 | 2.43E-61 |
| PPP1R2 | 1.48E-64 | 1.624614 | 4.69E-61 |
| YPEL5 | 1.71E-64 | 1.290282 | 5.42E-61 |
| CFD | 3.66E-64 | -1.63013 | 1.16E-60 |
| MAFB | 6.44E-64 | -1.10201 | 2.04E-60 |
| CD48 | 3.78E-63 | 1.317867 | 1.20E-59 |
| IFI30 | 5.59E-63 | -1.03756 | 1.77E-59 |
| EMP3 | 9.35E-63 | -1.01863 | 2.96E-59 |
| SOD1 | 1.19E-62 | 1.277764 | 3.77E-59 |
| PLD3 | 2.17E-61 | -1.05576 | 6.87E-58 |
| THBD | 5.08E-60 | -1.09206 | 1.61E-56 |
| SRSF7 | 1.18E-59 | 1.145323 | 3.72E-56 |
| FTH1 | 1.90E-59 | -1.26022 | 6.00E-56 |
| FCGR3A | 2.94E-59 | -1.48553 | 9.28E-56 |
| ITGB2 | 3.26E-59 | -1.07384 | 1.03E-55 |
| ISG20 | 6.84E-59 | 1.153337 | 2.16E-55 |
| NAMPT | 1.84E-58 | -1.10004 | 5.81E-55 |
| LINC01272 | 8.95E-58 | -1.14798 | 2.83E-54 |
| CXCL3 | 1.84E-57 | -2.22815 | 5.81E-54 |
| STK4 | 3.10E-57 | 1.315748 | 9.79E-54 |
| CYB5A | 3.97E-57 | -1.22446 | 1.26E-53 |
| TSC22D1 | 6.62E-55 | -1.34619 | 2.09E-51 |
| EGR1 | 6.99E-55 | -1.35508 | 2.21E-51 |
| CD59 | 6.91E-54 | -1.05082 | 2.18E-50 |
| DUSP1 | 1.42E-53 | -1.06451 | 4.49E-50 |
| EREG | 6.36E-53 | -2.12777 | 2.01E-49 |
| JUNB | 2.38E-52 | 1.09007 | 7.54E-49 |
| TUBA4A | 1.44E-51 | 1.536566 | 4.56E-48 |
| TGM2 | 1.00E-50 | -1.02161 | 3.17E-47 |
| CACYBP | 1.13E-49 | 1.185958 | 3.56E-46 |
| FAM177A1 | 1.68E-49 | 1.431856 | 5.30E-46 |
| S100A8 | 6.33E-48 | -1.55229 | 2.00E-44 |
| VCAN | 9.42E-47 | -1.16757 | 2.98E-43 |
| BCL2A1 | 1.18E-46 | -1.18909 | 3.73E-43 |
| HSPB1 | 1.36E-46 | -1.09885 | 4.30E-43 |
| LY6E | 3.80E-46 | -1.12661 | 1.20E-42 |
| MGST1 | 4.69E-46 | -1.40988 | 1.48E-42 |
| ELF1 | 1.44E-45 | 1.197621 | 4.57E-42 |
| SRSF5 | 1.69E-45 | 1.066108 | 5.33E-42 |
| CYTIP | 1.47E-44 | 1.110128 | 4.65E-41 |
| EMP2 | 3.94E-44 | -1.19969 | 1.24E-40 |
| ACP5 | 1.54E-43 | -1.27733 | 4.87E-40 |
| FOS | 1.83E-43 | -1.18554 | 5.79E-40 |
| ALOX5AP | 3.05E-43 | -1.43318 | 9.65E-40 |
| SOX4 | 8.96E-43 | -1.3407 | 2.83E-39 |
| IL1B | 1.24E-41 | -2.13323 | 3.91E-38 |
| SOD2 | 4.67E-40 | -1.24081 | 1.48E-36 |
| MCEMP1 | 5.06E-40 | -1.09372 | 1.60E-36 |
| SC5D | 1.08E-39 | 1.407204 | 3.41E-36 |
| ELF3 | 8.57E-37 | -1.86739 | 2.71E-33 |
| PNISR | 2.69E-36 | 1.004559 | 8.52E-33 |
| FCN1 | 5.21E-36 | -1.41041 | 1.65E-32 |
| CD37 | 8.37E-36 | 1.047431 | 2.65E-32 |
| C8orf4 | 4.28E-35 | -1.33055 | 1.35E-31 |
| NAPSA | 4.74E-35 | -1.70592 | 1.50E-31 |
| PPP2R5C | 5.16E-35 | 1.276328 | 1.63E-31 |
| RETN | 5.62E-35 | -1.24717 | 1.78E-31 |
| TM4SF1 | 2.97E-34 | -1.81137 | 9.39E-31 |
| SFTPB | 5.00E-34 | -2.10156 | 1.58E-30 |
| SPP1 | 1.81E-33 | -2.75925 | 5.73E-30 |
| ZFP36L2 | 1.70E-32 | 1.237785 | 5.36E-29 |
| MT2A | 5.36E-32 | -1.73714 | 1.69E-28 |
| KRT8 | 6.07E-32 | -1.1409 | 1.92E-28 |
| CCL3 | 8.83E-32 | -1.75157 | 2.79E-28 |
| SFTA2 | 1.09E-30 | -1.25652 | 3.45E-27 |
| IL32 | 1.59E-30 | 1.272003 | 5.04E-27 |
| LEPROTL1 | 2.42E-29 | 1.264526 | 7.66E-26 |
| KRT18 | 1.25E-28 | -1.29353 | 3.96E-25 |
| WFDC2 | 8.17E-28 | -1.70407 | 2.58E-24 |
| DDIT4 | 4.85E-27 | 1.056665 | 1.53E-23 |
| RP11-138A9.1 | 7.62E-27 | 1.047866 | 2.41E-23 |
| EVL | 1.63E-25 | 1.127357 | 5.14E-22 |
| KRT19 | 4.55E-25 | -1.02472 | 1.44E-21 |
| SSR4 | 5.89E-23 | -1.16259 | 1.86E-19 |
| IDS | 3.14E-21 | 1.076555 | 9.93E-18 |
| SFTPA1 | 7.90E-18 | -1.47613 | 2.50E-14 |
| PDCD4 | 8.69E-18 | 1.000734 | 2.75E-14 |
| ODF2L | 2.81E-16 | 1.112697 | 8.89E-13 |
| SFTPA2 | 3.11E-16 | -1.16757 | 9.82E-13 |
| PMAIP1 | 1.17E-15 | 1.124409 | 3.70E-12 |
| DUSP4 | 3.29E-13 | 1.117392 | 1.04E-09 |
| HOPX | 4.90E-12 | -1.77146 | 1.55E-08 |
| NR3C1 | 3.01E-08 | 1.097201 | 9.51E-05 |

**Supplementary Table 2. Variance analysis results of 213 genes in the branch II based on sc-RNA seq.**

| Gene | P value | log_2_FC | P value-adjusted |
| --- | --- | --- | --- |
| TYROBP | 2.25E-293 | 4.418816 | 7.11E-290 |
| AIF1 | 4.13E-293 | 3.258842 | 1.31E-289 |
| FCER1G | 1.17E-282 | 3.599519 | 3.71E-279 |
| LST1 | 4.30E-282 | 2.862809 | 1.36E-278 |
| HLA-DPA1 | 6.28E-260 | 3.756188 | 1.99E-256 |
| CST3 | 4.40E-255 | 3.300396 | 1.39E-251 |
| HLA-DRA | 3.87E-249 | 3.718448 | 1.22E-245 |
| HLA-DRB1 | 4.94E-248 | 3.672168 | 1.56E-244 |
| LYZ | 2.08E-244 | 5.900004 | 6.57E-241 |
| HLA-DPB1 | 1.62E-242 | 3.506756 | 5.12E-239 |
| MS4A6A | 2.81E-242 | 2.84049 | 8.89E-239 |
| MS4A7 | 2.17E-239 | 2.628357 | 6.87E-236 |
| C1orf162 | 2.53E-238 | 2.468028 | 8.00E-235 |
| GPX1 | 1.87E-235 | 2.542437 | 5.92E-232 |
| CD74 | 2.65E-233 | 3.067688 | 8.37E-230 |
| CD68 | 6.35E-232 | 3.027139 | 2.01E-228 |
| HLA-DQA1 | 4.76E-230 | 3.264462 | 1.50E-226 |
| OLR1 | 6.46E-226 | 2.64155 | 2.04E-222 |
| FCGR2A | 5.05E-223 | 2.444681 | 1.60E-219 |
| HLA-DQB1 | 2.21E-222 | 3.097757 | 6.97E-219 |
| CSTA | 1.67E-219 | 2.337054 | 5.29E-216 |
| GRN | 1.17E-215 | 2.794476 | 3.68E-212 |
| HLA-DMA | 2.63E-212 | 2.254709 | 8.30E-209 |
| HLA-DMB | 3.90E-208 | 2.261906 | 1.23E-204 |
| FTL | 1.18E-198 | 3.115104 | 3.74E-195 |
| CTSB | 2.90E-198 | 2.65789 | 9.17E-195 |
| PLAUR | 1.27E-196 | 2.725321 | 4.01E-193 |
| RAB31 | 4.45E-191 | 1.981271 | 1.41E-187 |
| SERPINA1 | 5.10E-188 | 2.012091 | 1.61E-184 |
| FCGRT | 2.76E-181 | 2.009473 | 8.73E-178 |
| CLEC7A | 1.42E-179 | 1.482146 | 4.49E-176 |
| NCF2 | 1.26E-177 | 1.537363 | 3.98E-174 |
| CXCL16 | 1.17E-173 | 1.876981 | 3.71E-170 |
| MNDA | 5.67E-172 | 1.685657 | 1.79E-168 |
| PSAP | 3.13E-169 | 2.466312 | 9.90E-166 |
| GLUL | 1.23E-163 | 2.281325 | 3.89E-160 |
| CAPG | 3.22E-162 | 2.011248 | 1.02E-158 |
| SLC11A1 | 4.49E-162 | 1.968761 | 1.42E-158 |
| CTSH | 2.84E-160 | 1.640936 | 8.98E-157 |
| MRC1 | 3.60E-158 | 1.923825 | 1.14E-154 |
| TGFBI | 5.17E-156 | 1.626143 | 1.63E-152 |
| ITGB2 | 8.43E-156 | 1.636509 | 2.66E-152 |
| NPC2 | 2.73E-155 | 1.48394 | 8.63E-152 |
| FTH1 | 1.26E-150 | 1.680288 | 3.98E-147 |
| IGSF6 | 4.95E-149 | 1.447973 | 1.57E-145 |
| PLEK | 3.75E-148 | 1.534504 | 1.19E-144 |
| H2AFY | 2.23E-147 | 1.46036 | 7.06E-144 |
| ALOX5AP | 2.02E-146 | 2.150048 | 6.40E-143 |
| RPS27A | 4.95E-146 | -1.05323 | 1.57E-142 |
| LY86 | 5.19E-146 | 1.33113 | 1.64E-142 |
| PPT1 | 3.97E-145 | 1.589764 | 1.25E-141 |
| CTSS | 6.66E-145 | 1.908306 | 2.11E-141 |
| MS4A4A | 1.30E-144 | 1.755807 | 4.12E-141 |
| ALOX5 | 2.88E-144 | 1.240243 | 9.09E-141 |
| RPL3 | 1.03E-143 | -1.10267 | 3.25E-140 |
| RNASE6 | 1.32E-143 | 1.601821 | 4.18E-140 |
| C5AR1 | 7.16E-143 | 1.668957 | 2.26E-139 |
| RPS26 | 4.45E-141 | -1.06779 | 1.41E-137 |
| CYBB | 5.19E-141 | 1.404483 | 1.64E-137 |
| ALDH2 | 6.02E-140 | 1.912323 | 1.90E-136 |
| RPS3 | 1.66E-138 | -1.03714 | 5.25E-135 |
| TMSB10 | 1.76E-138 | 1.179428 | 5.56E-135 |
| MGAT1 | 1.82E-138 | 1.530849 | 5.75E-135 |
| PHACTR1 | 1.87E-138 | 1.555833 | 5.92E-135 |
| S100A11 | 2.11E-138 | 1.602297 | 6.67E-135 |
| ACTB | 6.69E-138 | 1.477021 | 2.11E-134 |
| CPVL | 1.22E-134 | 1.566505 | 3.84E-131 |
| KYNU | 2.10E-134 | 1.319342 | 6.65E-131 |
| CD86 | 2.43E-134 | 1.199538 | 7.67E-131 |
| CTSZ | 9.91E-134 | 1.491031 | 3.13E-130 |
| AP2S1 | 1.87E-131 | 1.428333 | 5.92E-128 |
| ARPC3 | 8.62E-131 | 1.247549 | 2.73E-127 |
| BCL2A1 | 1.95E-129 | 1.779093 | 6.15E-126 |
| FGR | 9.52E-127 | 1.083577 | 3.01E-123 |
| SLC7A7 | 1.17E-126 | 1.27024 | 3.70E-123 |
| SLC16A10 | 3.58E-126 | 1.665392 | 1.13E-122 |
| GSN | 5.35E-126 | 1.52147 | 1.69E-122 |
| ATP6V1F | 1.85E-125 | 1.428762 | 5.84E-122 |
| IFI30 | 2.40E-125 | 1.413758 | 7.59E-122 |
| PILRA | 7.90E-125 | 1.112015 | 2.50E-121 |
| PLSCR1 | 3.22E-124 | 1.256707 | 1.02E-120 |
| FCGR3A | 4.87E-123 | 1.950784 | 1.54E-119 |
| CSF1R | 1.90E-122 | 1.09376 | 6.02E-119 |
| MFSD1 | 3.53E-122 | 1.105285 | 1.12E-118 |
| LGALS1 | 1.27E-121 | 1.400789 | 4.01E-118 |
| VIM | 1.56E-121 | 1.239314 | 4.93E-118 |
| PLBD1 | 8.33E-121 | 1.149156 | 2.63E-117 |
| RNF130 | 2.05E-120 | 1.1551 | 6.49E-117 |
| KLF4 | 1.09E-118 | 1.485877 | 3.44E-115 |
| CTSC | 2.94E-118 | 1.956418 | 9.30E-115 |
| HAVCR2 | 4.68E-118 | 1.092675 | 1.48E-114 |
| RP11-1143G9.4 | 7.23E-118 | 2.572846 | 2.29E-114 |
| PYCARD | 4.13E-117 | 1.291006 | 1.31E-113 |
| ACSL1 | 7.91E-117 | 1.319373 | 2.50E-113 |
| G0S2 | 9.25E-117 | 3.823732 | 2.92E-113 |
| LAPTM5 | 5.49E-115 | 1.264538 | 1.74E-111 |
| TSPO | 1.41E-114 | 1.57116 | 4.45E-111 |
| THBS1 | 7.16E-114 | 2.106571 | 2.26E-110 |
| ATP6V0B | 5.96E-112 | 1.32805 | 1.88E-108 |
| S100A9 | 1.00E-111 | 1.8641 | 3.17E-108 |
| COTL1 | 3.99E-111 | 1.12514 | 1.26E-107 |
| LAIR1 | 1.83E-110 | 1.029714 | 5.80E-107 |
| ASAH1 | 3.97E-110 | 1.547448 | 1.25E-106 |
| PFN1 | 2.05E-108 | 1.110806 | 6.49E-105 |
| SAT1 | 6.54E-108 | 1.318768 | 2.07E-104 |
| APOC1 | 1.21E-107 | 3.898753 | 3.82E-104 |
| VAMP8 | 1.67E-105 | 1.090578 | 5.27E-102 |
| CTSD | 6.06E-105 | 2.830881 | 1.91E-101 |
| ANXA5 | 1.93E-104 | 1.197347 | 6.11E-101 |
| C15orf48 | 4.92E-104 | 2.133498 | 1.56E-100 |
| LIMS1 | 4.82E-103 | 1.377447 | 1.53E-99 |
| ATP6V1B2 | 6.42E-102 | 1.104274 | 2.03E-98 |
| TREM1 | 1.75E-101 | 1.346578 | 5.53E-98 |
| RAB20 | 1.82E-101 | 1.214818 | 5.75E-98 |
| LINC01272 | 6.97E-101 | 1.429172 | 2.20E-97 |
| ARHGAP18 | 7.30E-101 | 1.019203 | 2.31E-97 |
| CYBA | 1.49E-100 | 1.053918 | 4.73E-97 |
| SH3BGRL3 | 1.25E-99 | 1.173055 | 3.96E-96 |
| BASP1 | 3.52E-99 | 1.171733 | 1.11E-95 |
| ITGAX | 4.38E-99 | 1.049829 | 1.39E-95 |
| LILRB4 | 9.31E-99 | 1.003715 | 2.94E-95 |
| MAFB | 4.86E-98 | 1.312971 | 1.54E-94 |
| EMP3 | 5.24E-96 | 1.197665 | 1.66E-92 |
| KCTD12 | 5.29E-96 | 1.001298 | 1.67E-92 |
| BLOC1S1 | 3.22E-94 | 1.053537 | 1.02E-90 |
| HEXB | 5.05E-93 | 1.054095 | 1.60E-89 |
| GSTO1 | 7.56E-93 | 1.267756 | 2.39E-89 |
| UPP1 | 3.57E-92 | 1.027218 | 1.13E-88 |
| PTPRE | 4.23E-92 | 1.146209 | 1.34E-88 |
| EREG | 1.90E-91 | 2.515407 | 6.02E-88 |
| HBEGF | 2.85E-91 | 1.431292 | 9.00E-88 |
| AVPI1 | 3.89E-91 | 1.225697 | 1.23E-87 |
| PMP22 | 4.04E-91 | 1.319688 | 1.28E-87 |
| ITM2B | 1.01E-90 | 1.216147 | 3.18E-87 |
| ARPC1B | 2.12E-90 | 1.077268 | 6.70E-87 |
| S100A4 | 4.19E-90 | 1.397258 | 1.32E-86 |
| ETS2 | 1.51E-89 | 1.010479 | 4.76E-86 |
| FBP1 | 2.09E-89 | 1.703967 | 6.62E-86 |
| THBD | 2.14E-89 | 1.17104 | 6.78E-86 |
| RNF13 | 8.09E-89 | 1.032173 | 2.56E-85 |
| CLEC5A | 1.47E-88 | 1.021862 | 4.65E-85 |
| SLC16A3 | 1.61E-88 | 1.009454 | 5.09E-85 |
| S100A8 | 1.92E-88 | 1.903111 | 6.06E-85 |
| ARPC5 | 1.48E-86 | 1.041389 | 4.67E-83 |
| RGS10 | 1.04E-85 | 1.101932 | 3.30E-82 |
| IER3 | 1.21E-85 | 1.593202 | 3.83E-82 |
| HMOX1 | 3.88E-85 | 1.155305 | 1.23E-81 |
| CXCL8 | 2.65E-84 | 2.765287 | 8.37E-81 |
| CFD | 1.02E-83 | 1.747441 | 3.23E-80 |
| ANXA2 | 3.48E-83 | 1.038934 | 1.10E-79 |
| DAB2 | 3.10E-82 | 1.065764 | 9.79E-79 |
| ACP5 | 8.86E-82 | 1.672567 | 2.80E-78 |
| CTSL | 2.12E-81 | 1.819072 | 6.70E-78 |
| IL1B | 3.16E-81 | 2.570961 | 1.00E-77 |
| CD83 | 4.97E-80 | 1.029394 | 1.57E-76 |
| SGK1 | 1.08E-79 | 1.365939 | 3.42E-76 |
| HEXA | 9.84E-79 | 1.000517 | 3.11E-75 |
| FNIP2 | 3.18E-77 | 1.15236 | 1.00E-73 |
| CSTB | 1.51E-76 | 1.453894 | 4.79E-73 |
| CD63 | 1.06E-74 | 1.052865 | 3.37E-71 |
| CD14 | 1.61E-74 | 1.077585 | 5.08E-71 |
| MCEMP1 | 6.93E-73 | 1.39017 | 2.19E-69 |
| NAMPT | 4.83E-70 | 1.163764 | 1.53E-66 |
| ATP1B3 | 4.84E-70 | 1.100027 | 1.53E-66 |
| GPR183 | 2.60E-69 | 1.43486 | 8.21E-66 |
| NINJ1 | 5.60E-69 | 1.064136 | 1.77E-65 |
| STXBP2 | 7.65E-69 | 1.045363 | 2.42E-65 |
| TSC22D3 | 8.33E-68 | -1.52917 | 2.63E-64 |
| FCN1 | 8.20E-67 | 1.750211 | 2.59E-63 |
| BIRC3 | 1.34E-66 | -1.6054 | 4.23E-63 |
| NR4A3 | 6.32E-66 | 1.124534 | 2.00E-62 |
| RETN | 1.50E-65 | 1.558969 | 4.75E-62 |
| LGALS3 | 6.74E-65 | 1.275109 | 2.13E-61 |
| VCAN | 2.10E-64 | 1.061587 | 6.65E-61 |
| FAM177A1 | 2.51E-63 | -1.74234 | 7.92E-60 |
| SOD2 | 1.51E-61 | 1.473183 | 4.78E-58 |
| CCL3 | 3.09E-61 | 2.32986 | 9.78E-58 |
| IER5 | 1.10E-58 | 1.004329 | 3.47E-55 |
| CXCL3 | 2.01E-53 | 2.196015 | 6.35E-50 |
| TUBA4A | 5.88E-53 | -1.55435 | 1.86E-49 |
| SOD1 | 4.78E-52 | -1.20696 | 1.51E-48 |
| IFI6 | 2.14E-49 | 1.321054 | 6.76E-46 |
| CD9 | 3.33E-49 | 1.003726 | 1.05E-45 |
| ISG20 | 5.58E-49 | -1.41704 | 1.76E-45 |
| IL32 | 5.55E-48 | -1.50347 | 1.75E-44 |
| SRSF7 | 1.31E-46 | -1.11826 | 4.15E-43 |
| FABP5 | 2.48E-46 | 1.288551 | 7.85E-43 |
| CXCL2 | 4.19E-45 | 1.376204 | 1.32E-41 |
| ARID5B | 3.49E-40 | -1.50024 | 1.10E-36 |
| CACYBP | 5.08E-39 | -1.11335 | 1.61E-35 |
| OCIAD2 | 2.70E-34 | -1.17258 | 8.54E-31 |
| C12orf57 | 2.80E-33 | -1.02111 | 8.86E-30 |
| SC5D | 5.85E-33 | -1.22715 | 1.85E-29 |
| ODF2L | 7.61E-30 | -1.13386 | 2.41E-26 |
| PPP1R2 | 4.98E-27 | -1.26431 | 1.58E-23 |
| CD69 | 1.21E-26 | -1.73261 | 3.83E-23 |
| PLAC8 | 2.86E-26 | 1.095926 | 9.05E-23 |
| STAT3 | 3.94E-26 | -1.03176 | 1.25E-22 |
| CXCR4 | 4.11E-26 | -1.23293 | 1.30E-22 |
| DDIT4 | 1.78E-23 | -1.17544 | 5.62E-20 |
| BTG2 | 5.30E-23 | -1.04606 | 1.68E-19 |
| IL7R | 1.60E-22 | -1.79231 | 5.07E-19 |
| IRF1 | 4.95E-21 | -1.01709 | 1.56E-17 |
| PPP2R5C | 1.63E-20 | -1.01878 | 5.15E-17 |
| SPP1 | 2.19E-20 | 2.391493 | 6.94E-17 |
| HOPX | 7.95E-17 | -2.34849 | 2.51E-13 |
| TM4SF1 | 9.25E-17 | -1.72075 | 2.92E-13 |
| C8orf4 | 1.46E-16 | -1.23777 | 4.62E-13 |
| CCL20 | 5.06E-12 | 1.036017 | 1.60E-08 |
| KRT8 | 4.49E-10 | -1.0029 | 1.42E-06 |
| SFTA2 | 3.98E-08 | -1.10597 | 0.000125765 |
| KRT18 | 3.21E-06 | -1.18187 | 0.010142301 |
| WFDC2 | 6.84E-06 | -1.58176 | 0.021638854 |

**Supplementary Table 3. Univariate analysis results of 77 genes in the LUAD training cohort.**

| **Gene** | | | **HR** | | | | **HR.95L** | | | **HR.95H** | | | **P value** | | |
| --- | --- | --- | --- | --- | --- | --- | --- | --- | --- | --- | --- | --- | --- | --- | --- |
| **NPC2** | | | 0.797205 | | | | 0.687807 | | | 0.924003 | | | 0.002617 | | |
| **FCGRT** | | | 0.733017 | | | | 0.603485 | | | 0.890352 | | | 0.001745 | | |
| **S100A11** | | | 1.453751 | | | | 1.130305 | | | 1.869754 | | | 0.003569 | | |
| **ALDH2** | | | 0.768305 | | | | 0.64485 | | | 0.915395 | | | 0.003187 | | |
| **PLAUR** | | | 1.224976 | | | | 1.053603 | | | 1.424222 | | | 0.008314 | | |
| **HLA-DMA** | | | 0.814572 | | | | 0.712409 | | | 0.931386 | | | 0.002704 | | |
| **ANXA2** | | | 1.410182 | | | | 1.125735 | | | 1.766503 | | | 0.002786 | | |
| **CD74** | | | 0.876481 | | | | 0.771214 | | | 0.996116 | | | 0.043427 | | |
| **ANXA5** | | | 1.369641 | | | | 1.023523 | | | 1.832804 | | | 0.034311 | | |
| **AP2S1** | | | 1.602446 | | | | 1.197609 | | | 2.144133 | | | 0.001505 | | |
| **HLA-DRA** | | | 0.887518 | | | | 0.789158 | | | 0.998138 | | | 0.046474 | | |
| **TIMP1** | | | 1.261577 | | | | 1.028492 | | | 1.547485 | | | 0.025779 | | |
| **ACTB** | | | 1.710137 | | | | 1.171127 | | | 2.497226 | | | 0.005474 | | |
| **IER3** | | | 1.169663 | | | | 1.019547 | | | 1.34188 | | | 0.025339 | | |
| **TGFBI** | | | 1.146109 | | | | 1.019566 | | | 1.288357 | | | 0.022338 | | |
| **HLA-DQB1** | | | 0.893223 | | | | 0.803774 | | | 0.992626 | | | 0.035955 | | |
| **HLA-DMB** | | | 0.868471 | | | | 0.76837 | | | 0.981613 | | | 0.024009 | | |
| **ASAH1** | | | 0.79806 | | | | 0.641924 | | | 0.992175 | | | 0.042287 | | |
| **HLA-DPB1** | | | 0.87881 | | | | 0.778902 | | | 0.991533 | | | 0.0359 | | |
| **KLF4** | | | 1.186371 | | | | 1.034909 | | | 1.36 | | | 0.014192 | | |
| **HLA-DQA1** | | | 0.88404 | | | | 0.793159 | | | 0.985333 | | | 0.025953 | | |
| **CLEC7A** | | | 0.851479 | | | | 0.739733 | | | 0.980106 | | | 0.025097 | | |
| **RAB13** | | | 1.581567 | | | | 1.068439 | | | 2.34113 | | | 0.021977 | | |
| **CSTB** | | | 1.294907 | | | | 1.01962 | | | 1.64452 | | | 0.034065 | | |
| **TXN** | | | 1.262909 | | | | 1.04294 | | | 1.529271 | | | 0.016823 | | |
| **PLSCR1** | | | 1.322308 | | | | 1.100389 | | | 1.588982 | | | 0.002877 | | |
| **BIRC3** | | | 1.269263 | | | | 1.119739 | | | 1.438754 | | | 0.000193 | | |
| **BLVRB** | | | 1.225529 | | | | 1.018771 | | | 1.474248 | | | 0.030988 | | |
| **S100A10** | | | 1.329097 | | | | 1.112263 | | | 1.588202 | | | 0.001743 | | |
| **HSPA8** | | | 1.25875 | | | | 1.002353 | | | 1.580732 | | | 0.047683 | | |
| **CD69** | | | 0.858717 | | | | 0.745017 | | | 0.989769 | | | 0.035564 | | |
| **LIMS1** | | | 1.391805 | | | | 1.061354 | | | 1.825143 | | | 0.016824 | | |
| **PKM** | | | 1.811165 | | | | 1.365371 | | | 2.40251 | | | 3.78E-05 | | |
| **FBP1** | | | 0.769834 | | | | 0.663043 | | | 0.893826 | | | 0.000597 | | |
| **ARPC1B** | | | 1.358599 | | | | 1.046956 | | | 1.763008 | | | 0.02116 | | |
| **GSTO1** | | | 1.315612 | | | | 1.023041 | | | 1.691854 | | | 0.032559 | | |
| **AVPI1** | | | 1.255459 | | | | 1.055948 | | | 1.492665 | | | 0.009981 | | |
| **PPP1R2** | | | 1.400658 | | | | 1.029552 | | | 1.905531 | | | 0.03192 | | |
| **SOD1** | | | 1.473294 | | | | 1.107577 | | | 1.959768 | | | 0.007772 | | |
| **CYB5A** | | | 0.851245 | | | | 0.733223 | | | 0.988264 | | | 0.034431 | | |
| **EREG** | | | 1.073417 | | | | 1.007581 | | | 1.143555 | | | 0.028247 | | |
| **TUBA4A** | | | 1.300372 | | | | 1.068327 | | | 1.582819 | | | 0.008818 | | |
| **CACYBP** | | | 1.726412 | | | | 1.308894 | | | 2.277111 | | | 0.000111 | | |
| **VCAN** | | | 1.188101 | | | | 1.052342 | | | 1.341375 | | | 0.005369 | | |
| **SOD2** | | | 1.254597 | | | | 1.059364 | | | 1.485811 | | | 0.008584 | | |
| **FCN1** | | | 0.855187 | | | | 0.735436 | | | 0.994437 | | | 0.042109 | | |
| **CD37** | | | 0.862316 | | | | 0.745849 | | | 0.996969 | | | 0.045395 | | |
| **TM4SF1** | | | 1.169674 | | | | 1.0162 | | | 1.346327 | | | 0.02897 | | |
| **SFTPB** | | | 0.884234 | | | | 0.837626 | | | 0.933437 | | | 8.46E-06 | | |
| **MT2A** | | | 1.231783 | | | | 1.074292 | | | 1.412362 | | | 0.00282 | | |
| **KRT8** | | | 1.549367 | | | | 1.275117 | | | 1.882601 | | | 1.06E-05 | | |
| **KRT18** | | | 1.571938 | | | | 1.298055 | | | 1.903609 | | | 3.64E-06 | | |
| **WFDC2** | | | 0.846851 | | | | 0.770804 | | | 0.9304 | | | 0.000535 | | |
| **DDIT4** | | | 1.293518 | | | | 1.119994 | | | 1.493927 | | | 0.000462 | | |
| **KRT19** | | | 1.190127 | | | | 1.016094 | | | 1.393968 | | | 0.030935 | | |
| **SSR4** | | | 0.770849 | | | | 0.596551 | | | 0.996074 | | | 0.046587 | | |
| **PMAIP1** | | | 1.132897 | | | | 1.011876 | | | 1.268392 | | | 0.030404 | | |
| **HOPX** | | | 0.901584 | | | | 0.825016 | | | 0.985259 | | | 0.022142 | | |
| **KYNU** | | | 1.307057 | | | | 1.185307 | | | 1.441312 | | | 7.97E-08 | | |
| **PFN1** | | | 1.946279 | | | | 1.318595 | | | 2.872756 | | | 0.000802 | | |
| **SLC16A3** | | | 1.472567 | | | | 1.222593 | | | 1.773652 | | | 4.56E-05 | | |
| **ARPC5** | | | 1.571146 | | | | 1.153078 | | | 2.140791 | | | 0.004205 | | |
| **CD83** | | | 0.822291 | | | | 0.69049 | | | 0.97925 | | | 0.028148 | | |
| **BTG2** | | | 0.782663 | | | | 0.675437 | | | 0.906911 | | | 0.001115 | | |
| **CCL20** | | | 1.091602 | | | | 1.024333 | | | 1.163289 | | | 0.006917 | | |
| **LDHA** | | | 1.967151 | | | | 1.530718 | | | 2.52802 | | | 1.25E-07 | | |
| **SERPINB5** | | | 1.139006 | | | | 1.068895 | | | 1.213715 | | | 5.93E-05 | | |
| **KRT6A** | | | 1.172923 | | | | 1.108344 | | | 1.241265 | | | 3.39E-08 | | |
| **TFF1** | | | 1.062802 | | | | 1.010469 | | | 1.117844 | | | 0.018067 | | |
| **CYP24A1** | | | 1.096457 | | | | 1.023113 | | | 1.175059 | | | 0.009139 | | |
| **FGB** | | | 1.055102 | | | | 1.007617 | | | 1.104824 | | | 0.022433 | | |
| **FGA** | | | 1.071168 | | | | 1.017766 | | | 1.127372 | | | 0.008416 | | |
| **ABCC2** | | | 1.154554 | | | | 1.082518 | | | 1.231384 | | | 1.23E-05 | | |
| **CTAG1B** | | | 1.607637 | | | | 1.034561 | | | 2.498158 | | | 0.034769 | | |
| **IGFBP1** | | | 1.237629 | | | | 1.141115 | | | 1.342306 | | | 2.65E-07 | | |
| **CPS1** | | | 1.089768 | | | | 1.035457 | | | 1.146928 | | | 0.000981 | | |
| **NTS** | | | 1.099784 | | | | 1.040716 | | | 1.162205 | | | 0.000733 | | |
| **Supplementary Table 4. Lasso analysis results of 24 genes in the LUAD training cohort.** | | | | | | | | | | | | | |  |  |
| **Gene** | | | | **Coef** | | | | | | | | | |  |  |
| **ACTB** | | | | 0.172506 | | | | | | | | | |  |  |
| **ASAH1** | | | | -0.02305 | | | | | | | | | |  |  |
| **CLEC7A** | | | | -0.19503 | | | | | | | | | |  |  |
| **RAB13** | | | | 0.194696 | | | | | | | | | |  |  |
| **PLSCR1** | | | | 0.056175 | | | | | | | | | |  |  |
| **BIRC3** | | | | 0.079833 | | | | | | | | | |  |  |
| **CD69** | | | | -0.06217 | | | | | | | | | |  |  |
| **SOD1** | | | | 0.182045 | | | | | | | | | |  |  |
| **CACYBP** | | | | 0.114599 | | | | | | | | | |  |  |
| **VCAN** | | | | 0.070971 | | | | | | | | | |  |  |
| **TM4SF1** | | | | 0.02179 | | | | | | | | | |  |  |
| **SFTPB** | | | | -0.03372 | | | | | | | | | |  |  |
| **KRT8** | | | | 0.003446 | | | | | | | | | |  |  |
| **DDIT4** | | | | 0.070207 | | | | | | | | | |  |  |
| **SSR4** | | | | -0.02646 | | | | | | | | | |  |  |
| **KYNU** | | | | 0.077911 | | | | | | | | | |  |  |
| **PFN1** | | | | 0.039552 | | | | | | | | | |  |  |
| **CCL20** | | | | 0.052838 | | | | | | | | | |  |  |
| **LDHA** | | | | 0.154715 | | | | | | | | | |  |  |
| **KRT6A** | | | | 0.0516 | | | | | | | | | |  |  |
| **ABCC2** | | | | 0.007405 | | | | | | | | | |  |  |
| **CTAG1B** | | | | 0.496364 | | | | | | | | | |  |  |
| **IGFBP1** | | | | 0.072027 | | | | | | | | | |  |  |
| **CPS1** | | | | 0.010468 | | | | | | | | | |  |  |
|  | | | | | | | | | | | | | |  |  |
| **Supplementary Table 5. Univariate analysis of risk score and clinicopathological characteristics.** | | | | | | | | | | | | | | | |
|  | | **HR** | | | | **HR.95L** | | | **HR.95H** | | | **P value** | | | |
| **age** | | 0.977656 | | | | 0.525949 | | | 1.81731 | | | 0.943047 | | | |
| **gender** | | 1.048512 | | | | 0.765186 | | | 1.436746 | | | 0.768187 | | | |
| **stage** | | 1.651039 | | | | 1.424011 | | | 1.914261 | | | 3.07E-11 | | | |
| **tumor purity** | | 1.091951 | | | | 0.75121 | | | 1.587249 | | | 0.644839 | | | |
| **CCL20 mutation** | | 13.12422 | | | | 1.793222 | | | 96.05343 | | | 0.011244 | | | |
| **risk score** | | 1.370621 | | | | 1.289263 | | | 1.457114 | | | 5.66E-24 | | | |
| **Supplementary Table 6. Multivariate analysis of risk score and clinicopathological characteristics.** | | | | | | | | | | | | | | |  |
|  | **HR** | | | | **HR.95L** | | | **HR.95H** | | | **P value** | | | |  |
| **age** | 1.334777 | | | | 0.9662 | | | 1.843956 | | | 0.079874 | | | |  |
| **gender** | 1.015603 | | | | 0.735106 | | | 1.40313 | | | 0.925204 | | | |  |
| **stage** | 1.509893 | | | | 1.292232 | | | 1.764216 | | | 2.13E-07 | | | |  |
| **tumor purity** | 1.006143 | | | | 0.689641 | | | 1.4679 | | | 0.974649 | | | |  |
| **CCL20 mutation** | 10.66538 | | | | 1.436756 | | | 79.17162 | | | 0.020652 | | | |  |
| **risk score** | 1.150006 | | | | 1.109732 | | | 1.191741 | | | 1.54E-14 | | | |  |
